# A robust mean and variance test with application to high-dimensional phenotypes

**DOI:** 10.1007/s10654-021-00805-w

**Published:** 2021-10-15

**Authors:** James R. Staley, Frank Windmeijer, Matthew Suderman, Matthew S. Lyon, George Davey Smith, Kate Tilling

**Affiliations:** 1 MRC Integrative Epidemiology Unit, Population Health Sciences, Bristol Medical School, University of Bristol, Bristol, BS8 2BN UK; 2 Department of Statistics and Nuffield College, University of Oxford, Oxford, UK; 3 National Institute for Health Research Bristol Biomedical Research Centre, University of Bristol, Oakfield House, Bristol, BS8 2BN UK

**Keywords:** Variability test, Joint location-and-scale test, DNA methylation, ARIES, ALSPAC

## Abstract

Most studies of continuous health-related outcomes examine differences in mean levels (location) of the outcome by exposure. However, identifying effects on the variability (scale) of an outcome, and combining tests of mean and variability (location-and-scale), could provide additional insights into biological mechanisms. A joint test could improve power for studies of high-dimensional phenotypes, such as epigenome-wide association studies of DNA methylation at CpG sites. One possible cause of heterogeneity of variance is a variable interacting with exposure in its effect on outcome, so a joint test of mean and variability could help in the identification of effect modifiers. Here, we review a scale test, based on the Brown-Forsythe test, for analysing variability of a continuous outcome with respect to both categorical and continuous exposures, and develop a novel joint location-and-scale score (JLSsc) test. These tests were compared to alternatives in simulations and used to test associations of mean and variability of DNA methylation with gender and gestational age using data from the Accessible Resource for Integrated Epigenomics Studies (ARIES). In simulations, the Brown-Forsythe and JLSsc tests retained correct type I error rates when the outcome was not normally distributed in contrast to the other approaches tested which all had inflated type I error rates. These tests also identified > 7500 CpG sites for which either mean or variability in cord blood methylation differed according to gender or gestational age. The Brown-Forsythe test and JLSsc are robust tests that can be used to detect associations not solely driven by a mean effect.

**Supplementary Information:**

The online version contains supplementary material available at 10.1007/s10654-021-00805-w.

## Introduction

Most investigations into health-related phenotypes have focused on determining whether an exposure affects the mean of a phenotype (location test). However, assessing whether an exposure affects the variability of a phenotype (scale test) could also provide insight into the biological mechanisms that control phenotypic variation and disease pathogenesis [1–3]. When the exposure is randomization within a randomized controlled trial (RCT), variance differences in the outcome can also be used to indicate the degree to which there is heterogeneity in response to treatment, and thus potential for improving treatment outcomes through patient stratification [4]. Analogously, variance differences by level of a genotype being employed as an instrumental variable within a Mendelian randomization [5] framework can provide evidence of violation of the assumption required for identification of an average treatment effect [6].

The potential of combining a location test with a scale test (joint location-and-scale test) has yet to be fully explored, especially in the context of high-dimensional phenotypes where these tests could be used to improve power. There are several ways in which an exposure could be associated with outcome variability, including: direct cause (exposure causes outcome variability); indirect cause (a common cause of exposure and outcome also causes outcome variability); interaction, where a third variable modifies the effect of exposure on outcome. Thus joint location-and-scale tests could be used to examine evidence for either a mechanistic effect or the existence of interactions [7], with further research needed to clarify exactly how the exposure affects the outcome variability. One example where these approaches could be particularly useful is for epigenome-wide association studies (EWAS), where DNA methylation at CpG (cytosine followed by a guanine) sites across the genome are tested for association with an exposure (Supplementary Text) [8, 9]. Differences in variability in methylation levels is key to much contemporary theorising in this area [1, 10].

A range of statistical tests have been developed to interrogate whether an exposure affects variability of an outcome, specifically in the context of evaluating variability differences for a continuous variable between groups of individuals [11]. Li et al. [12] compared approaches for assessing methylation variability in the EWAS setting, and showed that the Brown-Forsythe test [13] performed well compared to alternative approaches. Since this test can be re-formulated in a regression framework [14, 15], it can be extended to continuous exposures. Methods for jointly testing mean and variability have also been proposed [7, 14–19], although these approaches are either limited by sensitivity to distributional assumptions or are restricted to binary exposures.

Here we review variability tests, specifically the Brown-Forsythe test, and develop a novel joint location-and-scale test, which can be used for both continuous and categorical exposures. We performed a simulation study to compare these approaches to alternative tests, and then applied these modelling approaches to investigate the effect of gender and gestational age on cord blood DNA methylation mean and variability.

## Methods

### Modelling approaches

#### Location tests

Linear regression is commonly used to assess mean differences in methylation by an exposure. That is,

1 $$ y_{i}  = \alpha  +  x^{\prime}_{i} \beta  + \varepsilon _{i} ~,~i = 1, \ldots ,n~,~ $$

where ${y}_{i}$ is the outcome for the $i$-th individual (e.g. DNA methylation levels in epigenome-wide association studies), ${x}^{\prime}_{i}$ is the exposure(s) for the $i$-th individual and ${\epsilon }_{i}\sim N(0, {\sigma }_{\epsilon }^{2})$. A z-test can then be used to test the null hypothesis that the mean difference is zero (i.e. that $\beta$ =0). The assumption of Normality of the errors is necessary for correct finite sample inference (*p*-values of test statistics and confidence intervals)-however, for large sample sizes, non-normality of residuals (e.g. residuals following either a uniform or a beta distribution [20]) does not materially affect coverage (discussed further in the Supplementary Text). Alternatively, the variance sandwich estimator is robust to general forms of heteroskedasticity, i.e. when the variance of the ${\epsilon }_{i}$ varies with the values of ${x}_{i}$.

#### Scale tests

There are several statistical tests for assessing variability differences of continuous outcome by a categorical exposure [11]. Bartlett’s test [21] is perhaps the most well-known of these tests (Supplementary Text) and has been used to analyse high-dimensional phenotypes [3, 22]. However, this test is known to be very sensitive to outliers and non-normality of the outcome, which is a major cause of concern when analysing data like DNA methylation. The Brown-Forsythe test [13], on the other hand, has been shown in simulations to be relatively robust to non-normality of the outcome and outliers [12]. This test is essentially a one-way analysis of variability of the variable ${Z}_{j}=|{Y}_{j} -{M}_{j}|$, where ${Y}_{j}$ is the outcome (e.g. methylation) of the $j$-th category of exposure and ${M}_{j}$ is the population median outcome in the $j$-th category of exposure. Let ${Y}_{j}\sim N\left({\mu }_{j},{\sigma }_{j}^{2}\right)$, where ${\mu }_{j}$ and ${\sigma }_{j}^{2}$ are the population mean and variance of ${Y}_{j}$. Given a sample of *n* individuals from this population, the test statistic for ${H}_{0}:{\sigma }_{1}^{2}={\sigma }_{2}^{2}=\dots ={\sigma }_{k}^{2}$ is given by

2 $$ BF = { }\frac{{\left( {n - k} \right)\mathop \sum \nolimits_{j = 1}^{k} n_{j} \left( {\overline{z}_{j} - \overline{z}} \right)^{2} }}{{\left( {k - 1} \right)\mathop \sum \nolimits_{j = 1}^{k} \mathop \sum \nolimits_{i = 1}^{{n_{j} }} \left( {z_{ij} - \overline{z}_{j} } \right)^{2} }} , $$

where $k$ is the number of exposure categories, ${n}_{j}$ is the number of individuals in the sample in the $j$-th exposure category, ${z}_{ij}=|{y}_{ij} -{y}_{mj}|$, where ${y}_{ij}$ is the outcome for the $i$-th individual in the $j$-th category of exposure and ${y}_{mj}$ is the sample median outcome in the $j$-th category of exposure and ${\overline{z} }_{j}$ and $\overline{z }$ are the sample mean in the j-th category of exposure and overall sample mean of ${z}_{ij}$, respectively. Under the null hypothesis, ${H}_{0}:{\sigma }_{1}^{2}={\sigma }_{2}^{2}=\dots ={\sigma }_{k}^{2}$, $BF\sim {F}_{k-1.n-k}$.

We now consider data where the covariate is not (necessarily) categorical, i.e. instead of ${y}_{ij}$ being the outcome for the $i$-th individual in the $j$-th group (as above), we have ${y}_{i}$ as the outcome for the $i$-th individual, and ${x}_{i}$ as the value of the covariate for that individual. The Brown-Forsythe test can be re-formulated as a two-stage approach [14, 15]:

(i) Obtain the absolute values of the residuals from a least absolute deviation regression, ${d}_{i}= {|y}_{i}-(\widehat{\alpha }+ x^{\prime}_{i}\widehat{\beta })|$.
(ii) Test for an association between the ${d}_{i}$’s and a function of the ${x}^{\prime}_{i}$’s using a regression *F*-test.

To show that this reformulation is the same as (Eq. 2) in the case of a categorical covariate, note that the least absolute deviation regression predicted value $\widehat{\alpha }+ x^{\prime}_{i} \widehat{\beta }$ is the median of *y* in each category of the covariate. Thus, the absolute value of the residuals is the absolute value of deviations from the median, and thus regressing ${d}_{i}$ on ${x}_{i}$ gives the regression F-test of the same form as in (Eq. 2).

Since this regression framework does not depend on the exposure (${x}_{i}$) being categorical, it can also be applied to continuous exposures. Indeed, this approach has the same structure as the Glejser and Bresuch-Pagan tests of heteroskedasticity [23, 24].

#### Joint location-and-scale tests

If the outcome data are symmetrically distributed then the $p$-values from the location and scale tests are independent and can be combined using Fisher’s method (JLSp) [14, 15]. However, often high-dimensional phenotypes are not all symmetrically distributed (e.g. DNA methylation at CpG sites), which will likely lead to correlated $p$-values for at least some markers. Other alternative approaches for jointly testing for mean and variability effects include likelihood-ratio tests (LRT) comparing linear mixed models with and without including a fixed-effect and random-effect for the exposure (LRTmv) and double generalized linear mixed models (DGLM) [17, 18, 25] (further details in Supplementary Text). However, these tests are also sensitive to deviations from normality and outlying values [17].

To alleviate some of the issues involved in testing for mean and variability effects simultaneously, we have developed a joint location-and-scale score test (JLSsc). This approach essentially combines a location test and scale test, while accounting for the correlation between these tests.

For exposure X and outcome *Y*, we consider the conditional mean and variance specifications

$$ \begin{gathered} E\left( {Y|X = x} \right) = \alpha + x^{\prime}\beta \\ Var\left( {Y|X = x} \right) = \sigma^{2} \left( x \right) \\ \end{gathered} $$

and propose to test the joint null hypothesis $H_{0} :\beta = 0,\,\,\sigma^{2} \left( x \right) = \sigma^{2}$.

For a sample $\left\{ {y_{i} ,x^{\prime}_{i} } \right\}_{i = 1}^{n}$, the conditional linear model specification is then given by

3 $$ y_{i} = \alpha + x^{\prime}_{i} \beta + \varepsilon_{i} $$

with $E\left( {\varepsilon_{i} |x_{i} } \right) = 0$. The homoskedasticity restriction, $\sigma^{2} \left( x \right) = \sigma^{2}$, is commonly tested using the Breusch-Pagan auxiliary linear specification

4 $$ \hat{\varepsilon }_{i}^{2} = \gamma + x^{\prime}_{i} \delta + u_{i} , $$

where $\hat{\varepsilon }_{i}$ is the linear regression residual from (Eq. 3). The $nR^{2}$ from linear regression of(Eq. 4) is the score test for $H_{0} :\delta = 0$ in this linear specification, but which covers the null in the more general specification that $\sigma^{2} \left( x \right) = h\left( {\gamma + x^{\prime}_{i} \delta } \right)$ with $h\left( . \right)$ any positive function, for further details see the Supplementary Text.

The Breusch-Pagan score test only considers the specification under the null, as we do below, in which case $u_{i} = \hat{\varepsilon }_{i}^{2} - \gamma$, and the properties of $u_{i}$ under the null are therefore simply governed by the properties of the conditional moments of *Y* given *X*.

Our test procedure is to combine the linear model and auxiliary equations,

5 $$ \begin{gathered} y_{i} = \alpha + x^{\prime}_{i} \beta + \varepsilon_{i} \\ \left( {y_{i} - \overline{y}} \right)^{2} = \gamma + x^{\prime}_{i} \delta + u_{i} \\ \end{gathered} $$

where $\overline{y} = \frac{1}{n}\sum\limits_{i = 1}^{n} {y_{i} }$ is the sample mean of $y_{i}$, and test the joint null $H_{0} :\beta = \delta = 0$, which is a test for our general hypothesis $H_{0} :\beta = 0,\,\,\sigma^{2} \left( x \right) = \sigma^{2}$. For the variance specification test part we impose the restriction that $\beta = 0$, which enhances power.

For our variance estimator under the null to be consistent, as detailed below, we require that, under the null, the conditional skewness and kurtosis of *Y* given *X* are not a function of *X*. This assumption would automatically be satisfied when *Y* is normally distributed.

Let ${\tilde{y }}_{i}={y}_{i}-\overline{y }$, ${\tilde{x }}_{i}={x}_{i}-\overline{x }$ and ${\tilde{d }}_{i}={\tilde{y }}_{i}^{2}-{\widehat{\sigma }}^{2}$, where ${\widehat{\sigma }}^{2}=\frac{1}{n}{\sum }_{i=1}^{n}{\tilde{y }}_{i}^{2}$. Further, let the $n\times {k}_{x}$ matrix $\tilde{X} = \left[ {\tilde{x}_{i}^{'} } \right]$ and the $n$ vectors $\tilde{y }=\left({\tilde{y }}_{i}\right)$ and $\tilde{d }=\left({\tilde{d }}_{i}\right)$. Then the linear regression estimators for $\beta$ and $\delta$ in (Eq. 5) are given by

$$ \hat{\beta } = \left( {\tilde{X}^{\prime}\tilde{X}} \right)^{ - 1} \tilde{X}^{\prime}\tilde{y} $$

6 $$ \hat{\delta } = \left( {\tilde{X}^{\prime}\tilde{X}} \right)^{ - 1} \tilde{X}^{^{\prime}} \tilde{d}\, . $$

Let $$\theta =\left(\begin{array}{c}\beta \\ \delta \end{array}\right), \widehat{\theta }=\left(\begin{array}{c}\widehat{\beta }\\ \widehat{\delta }\end{array}\right)$$ and $$\widehat{\Sigma }=\frac{1}{n}\sum_{i=1}^{n}\left[\begin{array}{cc}{\tilde{y }}_{i}^{2}& {\tilde{y }}_{i}{\tilde{d }}_{i}\\ {\tilde{y }}_{i}{\tilde{d }}_{i}& {\tilde{d }}_{i}^{2}\end{array}\right]$$. A consistent estimator for the variance of $\widehat{\theta }$ under the null that $\beta =\delta =0$ and the additional assumption that the conditional skewness and kurtosis of $Y$ do not vary with the values of $X$, is then given by

7 $$ V\hat{a}r\left( {\hat{\theta }} \right) = {\hat{\Sigma }} \otimes \left( {\tilde{X}^{\prime}\tilde{X}} \right)^{ - 1} . $$

A test statistic for testing for ${H}_{0}:\beta =\delta =0$, or ${H}_{0}:\theta =0\,,$ is then given by

8 $$ S = \hat{\theta }^{\prime}\left( {{\hat{\Sigma }}^{ - 1} \otimes \left( {\tilde{X}^{\prime}\tilde{X}} \right)} \right)\hat{\theta } . $$

It follows from standard limiting distribution theory that, under the null, $S\stackrel{d}{\to }{\chi }_{2{k}_{x}}^{2}$. The proposed test using statistic *S* (Eq. 8) is a score test based on the joint asymptotic distribution of $\widehat{\beta }$ and $\widehat{\delta }$.

Additional terms such as the square of a continuous exposure, especially useful for modelling the relationship with outcome variability, can be added as part of ${x}_{i}$ vector and would be included in both parts of the test. Other variables that are expected to affect the outcome but are not considered important for testing purposes are regressed out of both the outcome and exposure variables by taking residuals from linear regression adjusting for these variables prior to analysis with JLSsc (see Supplementary Text). Further details of JLSsc are discussed in the Supplementary Text, including extensions for relaxing the conditional skewness and kurtosis assumption and a Brown-Forsythe formulation of the approach.

We have developed an R package to perform these tests available at: https://github.com/jrs95/jlst.

### Simulation study

We assessed the performance of the location and scale tests as well as the joint location-and-scale tests with both binary and continuous exposures in a simulation study based on methylation data. We assessed the performance of linear regression (estimated using ordinary least squares, OLS), Bartlett’s test (for simulations with a binary exposure), Brown-Forsythe test, LRT comparing mixed models with and without a variability effect (LRTv), JLSsc, JLSp, LRTmv and DGLM. For approaches which failed to adequately control type I error rates, we repeated the tests after applying M-value (i.e. ${\mathrm{log}}_{2}({y}_{i}/(1-{y}_{i}))$) [26] and inverse normal rank transformations to the methylation levels. This simulation study was performed based on data from the Tsaprouni et al*.* study [27], which investigated the relationship between smoking and DNA methylation (data accessible at NCBI GEO database [28], accession GSE50660).

Type I error simulations were performed by randomly generating a binary or continuous exposure (uncorrelated with mean or variability of any of the methylation levels) and testing the association of this exposure with mean and variability of DNA methylation at each CpG site in Tsaprouni *et al*. Although the distribution of DNA methylation at some CpG sites is highly skewed or has very thick tails, most have skewness between -1 and 1 (67.4%) and kurtosis less than 3 (74%). Histograms of the mean, standard deviation, skewness and kurtosis of all CpG sites are shown in Figure S1. To generate datasets with varying sample size (100, 500, 1000 and 10,000 samples), samples were taken with replacement from the Tsaprouni *et al.* dataset (Supplementary Text). The binary and continuous exposures were randomly generated using $Ber(0.5)$ and $N(\mathrm{0,1})$, respectively. Quantile–quantile (QQ) plots were used to assess deviations from normality and detect outlying test statistics.

Power simulations were performed using the same exposure distributions as above and setting these exposures to affect the mean and variability of methylation. In each simulation replicate, one CpG was selected at random from the Tsaprouni et al*.* dataset, the mean and standard deviation of this CpG site were used to set the average methylation and to generate mean and variability effects (Supplementary Text). The mean and variability effects of the exposure on methylation were simulated using normal distributions, while the residual error was simulated to be either normally distributed, heavy-tailed or skewed (Supplementary Text). Statistical power was calculated as the proportion of simulation replicates where either the location, scale or joint test had $p<1\times {10}^{-7}$. For each simulation scenario, 1000 simulation replicates were performed for a sample size of 1000 observations.

We also performed type I error and power simulations for a categorical exposure with three categories ($Bin$(2, 0.3)) and investigated adding a squared exposure term (i.e. the square of the simulated exposure) to the JLSsc approach in the continuous exposure (type I error and power) simulations (Supplementary Text). Additional power simulations were performed where we generated an outlying value (Supplementary Text).

The computational time of the extended Brown-Forsythe test and JLSsc were compared to their equivalent LRTs for 100,000 randomly selected CpGs from the Tsaprouni et al*.* dataset for the binary and continuous exposures describe above. This analysis was performed using one core (2.6 GHz; 4 GB) on a Linux server.

We set up further simulations to investigate type I error distributions, not using the Tsaprouni *et al.* dataset, but instead drawing the outcome variable distribution from a Normal (0, 1), a *t*-distribution with 4 degrees of freedom, a log-normal (0, 1) distribution or contaminated Normal 90% $N(0, \,1)$ & 10% $N(5,\, 1)$, and the exposure as a binary, three-category or a standard Normal variable (Supplementary Text).

### Application to offspring gender and gestational age on cord blood DNA methylation

#### Study population

This study used DNA methylation data generated as part of the Avon Longitudinal Study of Parents and Children (ALSPAC) [29, 30]. ALSPAC recruited 14,541 pregnant women with expected delivery dates between April 1991 and December 1992. Of these initial pregnancies, there were 14,062 live births and 13,988 children who were alive at 1 year of age. Please note that the study website contains details of all the data that is available through a fully searchable data dictionary and variable search tool (http://www.bristol.ac.uk/alspac/researchers/our-data/). Ethical approval for the study was obtained from the ALSPAC Ethics and Law Committee and the Local Research Ethics Committees. Informed consent for the use of data collected via questionnaires and clinics was obtained from participants following the recommendations of the ALSPAC Ethics and Law Committee at the time. Consent for biological samples has been collected in accordance with the Human Tissue Act (2004).

As part of the Accessible Resource for Integrated Studies (ARIES) project (http://www.ariesepigenomics.org.uk) [31], a sub-sample of 1018 ALSPAC child–mother pairs had DNA methylation measured. The ARIES participants were selected based on availability of DNA samples at two time-points for the mother (antenatal and at follow-up when the offspring was in adolescence) and at three time-points for the offspring (neonatal from cord blood, childhood (age 7) and adolescence (age 17)).

#### Laboratory methods, quality control and pre-processing

The laboratory methods and quality control procedures used have been described elsewhere [32]. In brief, the DNA methylation wet laboratory and pre-processing analyses were performed at the University of Bristol as part of the ARIES project, where the Infinium HumanMethylation450 BeadChip [33] was used to measure genome-wide DNA methylation levels at over 485,000 CpG sites. The methylation level at each CpG site was calculated as a beta value: the ratio of the methylated probe intensity and the overall intensity. These beta values range from 0 (no methylation) to 1 (complete methylation). The samples were processed using functional normalization with the meffil package [34, 35]. Further quality control procedures are described in the Supplementary Text.

### Statistical analysis

To investigate the mean and variability effects of gender and gestational age (in weeks, Supplementary Text) on cord blood methylation, we used the approaches which controlled type I error rates without transforming methylation levels, namely linear regression (estimated using ordinary least squares, OLS), the Brown-Forsythe test, JLSp and JLSsc. All analyses were adjusted for cell counts estimated using the method described by de Goede et al. for cord blood methylation [36]. We further adjusted for 20 surrogate variables to account for residual batch effects [37]. The gestational age analysis was further adjusted for offspring gender and whether the birth was by caesarean section as well as for maternal characteristics: age, smoking, pre-pregnancy BMI and weight, parity, education, family social class and alcohol intake during pregnancy. All these factors were included in all analysis models (i.e. in both stages of the variance and joint tests). CpGs were considered to be associated with either gender or gestational age if one of the location, scale or joint tests had $p<1\times {10}^{-7}$.

All analyses were performed using R (version 3.5.2).

## Results

### Simulation study

**Fig. 1:**
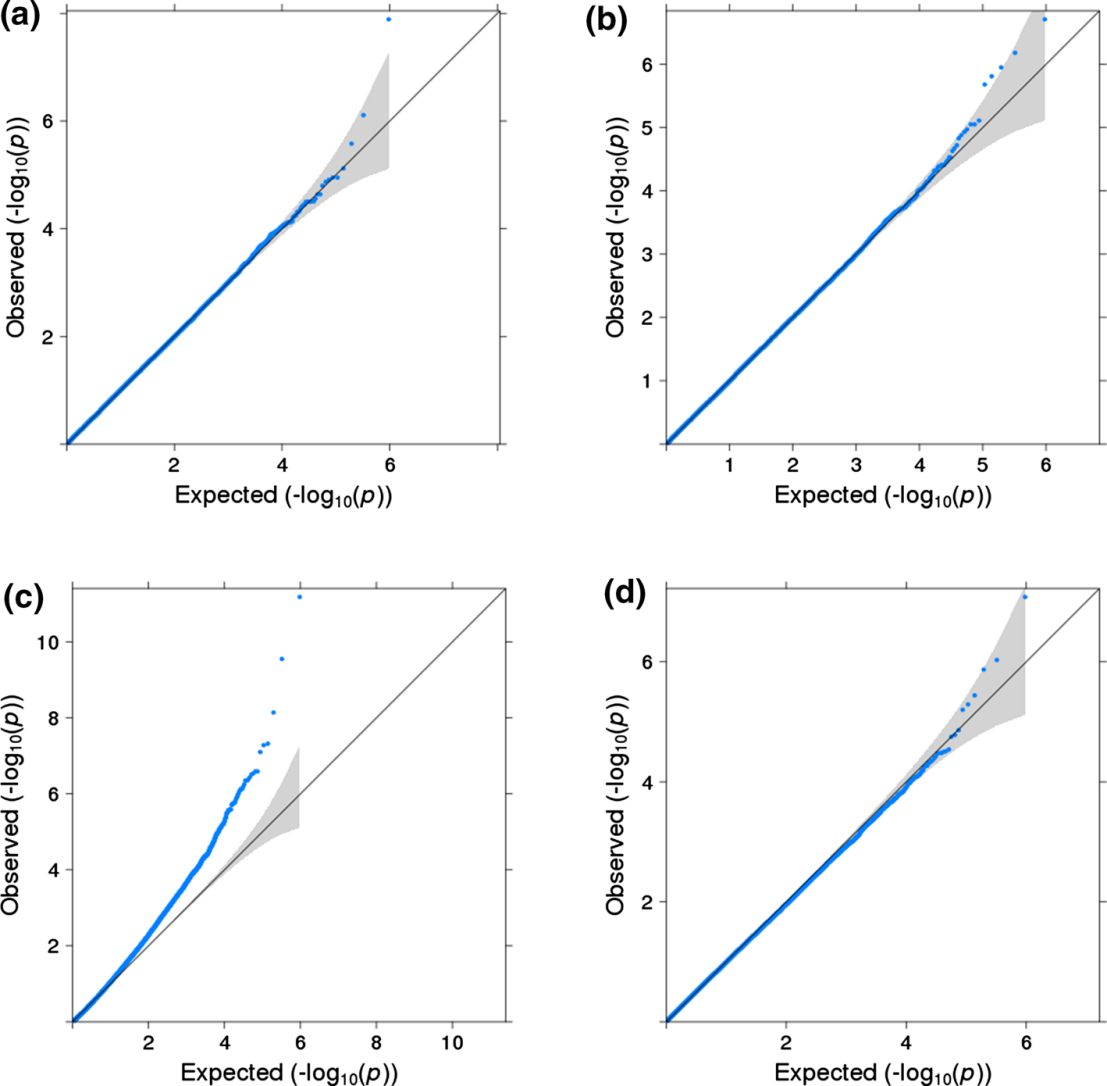
QQ plots for type I error simulations using a binary exposure and 1000 samples. **a** linear regression (mean test); **b** Brown-Forsythe (variability test); **c** JLSp (joint test); and **d** JLSsc (joint test)

The linear regression test (estimated using ordinary least squares, OLS) of mean differences was not inflated under the null of no mean or variability effect even in 100 samples (Figs. 1a and S2). Similarly, the Brown-Forsythe variability test accurately controlled type I error rates (Figs. 1b and S3). Bartlett’s test and LRTv had extreme type I error inflation due to the deviations from normality and the existence of outlying values in methylation levels (Figure S4). Likewise, the test statistics from the likelihood-based approaches for joint testing the mean and variability (LRTmv and DGLM) were also heavily inflated (Figure S4). The extreme inflated type I error rates of these approaches were still present after transforming methylation levels using the M-value transformation (Figure S5) but were no longer present after using an inverse normal rank transformation (Figure S6). However, when using this transformation a mean effect can induce a variability effect (Figure S7), as seen previously [38]. JLSp fared better than the aforementioned joint tests in controlling type I error rates, although the non-independence of the $p$-values did lead to a small amount of type I error inflation (Fig. 1c and S8). The JLSsc approach, on the other hand, correctly controlled type I error rates (Fig. 1d and S8).

**Fig. 2:**
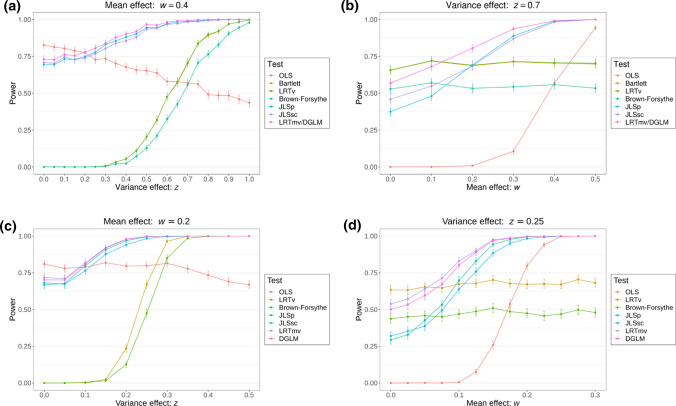
Power simulation results comparing approaches for identifying CpG sites associated with either a mean and/or a variance effect with the exposure at $p<1\times {10}^{-7}$. **a** & **b** are plots for a binary exposure and **c** & **d** are plots for a continuous exposure

In the power simulations, when there was either a mean or variability effect and the underlying data were normally distributed, the Brown-Forsythe test and JLSsc were less powerful but still performed well in comparison to the equivalent LRT and the alternative approaches (Fig. 2). This is expected as the Brown-Forsythe test and JLSsc sacrifice a small amount of power under the normal model for robustness to deviations from this model. Broadly similar results were found when the residual error was heavy-tailed or skewed, when the exposure was a categorical variable with three categories (although removing outliers [points > 3 standard deviations] in the outcome is necessary here to retain type I error levels), when a squared exposure term was added to JLSsc for a continuous exposure (likewise outlier removal in the outcome is necessary to retain type I error levels when testing both the exposure and the exposure-squared together), and when there was an outlier in the dataset (Figures S9-S13). The Brown-Forsythe formulation of the test also performed similarly in the scenarios tested (Supplementary Text and Figure S14). The additional type I error simulations (not drawing from existing methylation data) showed similar results-type I error was correctly controlled by JLSsc, with some inflation of error for JLSp when the mean and variance were not independent (e.g. when the outcome was drawn from log-normal or contaminated Normal distributions, Supplementary Text, Figures S15–S17 and Table S1).

The extension to JLSsc relaxing the constant skewness and kurtosis assumptions showed lower power than the usual version when the assumptions were met (Figure S18), and slightly less inflation of *p*-values when the assumptions were not met (Figure S19).

The computational time required to complete each approach for 100,000 CpGs with a binary exposure were as follows: 22 min for the extended Brown-Forsythe test, 113 min for LRTv, 16 min for JLSsc and 123 min for LRTmv. The relative computation times between the respective variability and joint tests were even greater when the exposure was continuous.

### Application to offspring gender and gestational age on cord blood DNA methylation

In ARIES, 858 children (417 male and 441 female) were available for the analysis of gender, and after excluding offspring with missing maternal information we were left with 708 children (345 males and 363 females) for the analysis of gestational age (mean: 39.5 weeks, standard deviation: 1.5 weeks; Table S2). Figures S20 and S21 show the distributions of skewness and kurtosis across methylation sites for the gender and gestational age analyses. These were broadly similar to those from the dataset used for the simulation study (Figure S1).

**Fig. 3:**
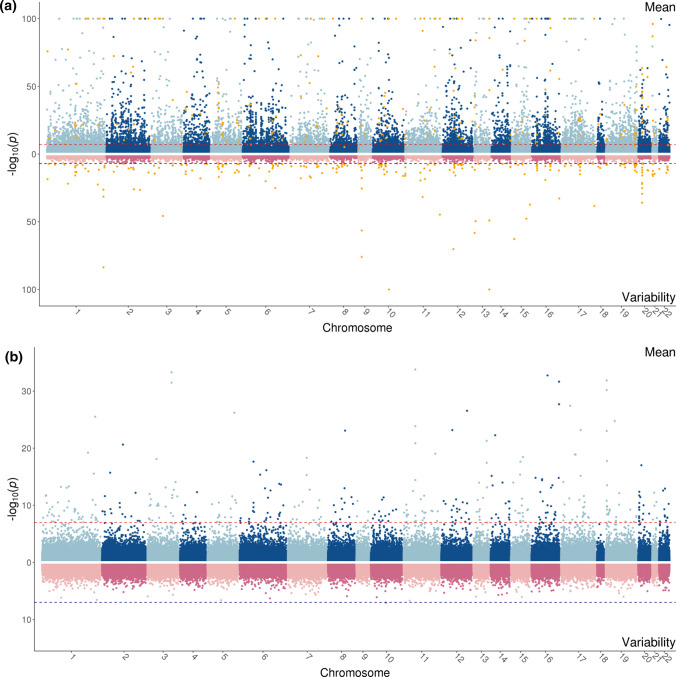
Miami plots for the mean (linear regression estimated using ordinary least squares, OLS) and variability (Brown-Forsythe test) associations of methylation with gender **a** and gestational age **b**. The dark red and blue lines represent the $p<1\times {10}^{-7}$ threshold and the orange points are CpG sites that are associated with a variance effect

**Fig. 4:**
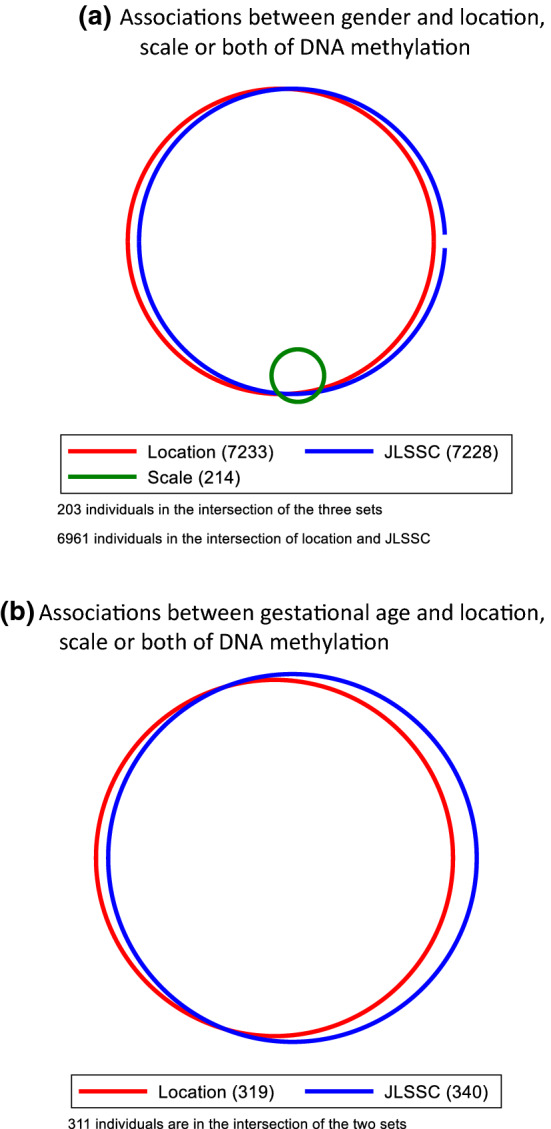
Venn diagrams showing the number of CPG sites identified as associated gestational age **a** or gender **b** by the location or scale test, or by JLSsc

Methylation at 8174 CpG sites were associated with gender in cord blood (through the mean, variability or joint tests; Fig. 3a and Table S3). Most of these sites were identified through a mean difference in methylation of males and females (7642 CpGs had a mean difference with$p<1\times {10}^{-7}$). 240 CpG sites were associated with a variability difference between males and females, of which all but 12 were also associated with a mean difference. For instance, cg18918831 was more variable in males compared to females (Figure S22). Using only the mean (regression) and variability (Brown-Forsythe) tests separately (taking account of the increasing number of tests done by using the cut-off $<5\times {10}^{-8}$) would have identified 7244 as having either a mean or a variability difference (or both). Of these, 6967 (96%) were identified by JLSsc which identified an additional 261 sites not identified by either location or scale tests (Fig. 4a). Results for JLSp are presented in Supplementary Text.

Mean methylation at 5359 of these sites were associated with gender in previous EWAS (Table S3) [39–42], corresponding to replication rates between 37–69% per EWAS. The highest rate was with the only other cord blood study [40], and the lowest rate with a study of adult peripheral blood [41]. Unexpectedly, however, replication rates above 54% were observed for a study of fetal brain [42] and peripheral blood in adults over the age of 70 [39]. Repeating the enrichment analyses of Singmann et al. [41], enrichments of the 8174 CpG sites were similarly observed in CpG island shores ($p < {2\times 10}^{-15}$; Fisher's exact test) and not among CpG sites annotated to genes with sex-hormone functions ($p > 0.4$). However, unlike Singmann et al., some enrichment was observed in CpG islands ($p < 5.2\times {10}^{-5}$), not at CpG sites annotated to imprinted genes ($p > 0.4$), and not for any Gene Ontology terms (Bonferroni-adjusted $p > 0.3$). There was no evidence for enrichment for the three enriched GO terms observed by Singmann *et al.* in adult blood ([41], nominal $p > 0.1$) nor any of the top ten enriched GO terms observed by Yousefi et al. in cord blood ([40], nominal $p > 0.2$). Enrichment methods and gene sets were identical to those previously described [41].

Gestational age was associated with cord blood methylation at 412 CpG sites (Fig. 3b and Table S4). Most of these CpG sites (354, 86%) were associated with a mean effect of gestational age on methylation, and there were no CpG sites with a variability effect with $p<1\times {10}^{-7}$. Using only the mean (regression) and variability (Brown-Forsythe) tests separately (taking account of the increasing number of tests done by using the cut-off $<5\times {10}^{-8}$) would have identified 319 CpG sites as having either a mean or a variability difference (or both), all of which had a mean effect. Of these, 311 (97%) were identified by JLSsc (Fig. 4b), which identified an additional 29 sites. Results for JLSp are presented in Supplementary Text. The majority of the CpG sites identified have been found previously in EWAS of gestational age (402 CpG sites; Table S4) [42, 43].

## Discussion

In this study, we have introduced a framework for testing variability using an extended version of the Brown-Forsythe test and for jointly testing mean and variability. These approaches were compared to the LRTs as well as other alternative methods in simulations and were used to investigate the effect of gender and gestational age on cord blood DNA methylation.

Without transforming the phenotype to be normally distributed, the approaches which assume normality of the phenotype (Bartlett’s test, LRTv, LRTmv and DGLM) had inflated type I error rates when faced with real methylation data. Indeed, these approaches essentially became tests of deviations from normality and outlying values, which can have some utility in identifying outliers caused by disease [44]. However, because of these drawbacks these approaches are not useful for assessing variability nor joint mean and variability effects. Normalizing outcome levels can overcome this problem, and give rise to nominally correct type-I error for the joint test. However, this transformation can induce mean or variability effects that were not present prior to the transformation, and thus may lead to erroneous conclusions about which of mean or variability effect (or both) was present [38]. In particular, kurtosis and skew of the untransformed variables (and how these relate to exposure) affect how mean and variability effects are induced by this transformation. The extended Brown-Forsythe test and the JLSsc approach retained correct type I error rates and performed well in comparison to the other approaches in detecting variability and joint effects. These tests were also at least 5 times more computationally efficient than their LRT counterparts.

Over 8000 CpG sites were associated with gender in cord blood methylation, while methylation at 412 CpG sites were associated with gestational age. The majority of these CpG sites were associated with effects of gender and gestational age on mean methylation. However, 240 CpG sites were associated with differences in variability between males and females. JLSsc identified most of the associations in both analyses, except where there was little evidence of a mean/variability effect in the presence of a borderline effect of the other. Although the main aim of this paper was to present and evaluate a method, we did briefly investigate how the 8174 sex-associated CpG sites compared to those identified in previous studies. Replication was high in spite of different tissues and ages at sample collection (37–69%). Like previous studies, we observed some similar enrichments in genomic regions, particularly CpG island shores. However, unlike previous studies, we did not observe any evidence for enrichment for any GO terms. In this particular example, nearly all sites captured by JLSsc are also captured by the mean-based test. However, there is no reason why we should expect this in general, i.e. for other phenotypes and exposures. Even for this example, the variability does indicate something of functional interest-that a small subset of the sites with mean differences also have variability differences. Contrast this with the gestational age example where no variance differences were discovered. Furthermore, of those sites with sex-specific variance, nearly two-thirds had greater variance in females. We applied Gene Ontology enrichment analysis to the differentially variable sites but found no evidence of enrichment after adjusting for multiple tests. The most enriched Gene Ontology terms from this analysis had very little overlap with the most enriched terms for the CpG sites with mean differences. This suggests that the functions of these differential variable CpG sites is likely quite different, however our findings do not support further speculation about the exact nature of these functions.

These methods are applicable to any area of medical research where variability and joint effects are of interest (e.g. comparing arms of a randomised controlled trial [4]), although they will be particularly useful for analysing high-dimensional phenotypes where it is not possible to assess the distribution at all markers. For instance, there has been recent interest in using variability tests to attempt to identify gene-environment interactions, as these interactions will often cause heterogeneity in the variance across genotypes [7, 38]. The Brown-Forsythe test has been proposed as a useful test in this scenario [38], although the extended version presented here and elsewhere [14, 15] could be used to assess variability trends across genotypes, which is also of value in assessing assumptions in Mendelian randomization, for example [6]. Furthermore, JLSsc avoids the distributional assumptions made by current methods proposed in the genetics literature [7, 14, 15].

The limitations of this study also warrant consideration. In the simulations and the applied example, we only analysed DNA methylation data, although we fully expect these results to be generalisable to all phenotypes. The application of the approaches to detect CpG sites associated with gestational age also have several limitations, especially with regards to residual confounding. In particular, there are likely to be other important maternal factors involved in gestation period that we have not adjusted for in our analysis. Where gender is the exposure there is unlikely to be residual confounding, but there may be effects of batch or cell count heterogeneity which remain. The ARIES cohort is also not selected at random from the full ALSPAC cohort [31], and as such, the results from this study may not generalise to the full ALSPAC cohort or the general population.

In summary, the extended Brown-Forsythe test and JLSsc are robust tests of variability and joint mean and variability effects, respectively. These tests can be used in analyses to detect associations for any type of exposure with high-dimensional phenotypes.

## Supplementary Information

Below is the link to the electronic supplementary material.

Supplementary file1 (XLSX 1154 kb)

Supplementary file2 (DOCX 14201 kb)

## Declarations

### Availability of data and material

All relevant data has been provided. ALSPAC data access is through a system of managed open access.

### Code availability

The R package to perform these tests is available at: https://github.com/jrs95/jlst.
