# Supplementary material for: A robust mean and variance test with application to high-dimensional phenotypes
Source: Eur J Epidemiol. 2021 Oct 15;37(4):377–87. doi: 10.1007/s10654-021-00805-w (PMC9187575; doi:10.1007/s10654-021-00805-w)

A robust mean and variance test with application to

high-dimensional phenotypes: Supplementary

James R Staley^1^, Frank Windmeijer^1,2^, Matthew Suderman^1^, Matthew S Lyon^1,3^, George Davey Smith^1^ and Kate Tilling^1,*^.

^1^MRC Integrative Epidemiology Unit, Population Health Sciences, Bristol Medical School, University of Bristol, Bristol, UK.

^2^Department of Statistics and Nuffield College, University of Oxford, Oxford, UK.

^3^National Institute for Health Research Bristol Biomedical Research Centre, University of Bristol, Oakfield House, Bristol, BS8 2BN, UK.

^*^Corresponding author.

Correspondence:

Prof Kate Tilling

MRC Integrative Epidemiology Unit, Population Health Sciences, Bristol Medical School, University of Bristol, Bristol, BS8 2BN, UK.

Email: kate.tilling@bristol.ac.uk

Telephone: +44 (0)117 331 0019

# Supplementary Text

## Epigenome-wide association studies

Epigenome-wide association studies (EWAS) have been used to assess associations of DNA methylation at CpG (cytosine followed by a guanine) sites from across the genome with diseases and traits [1, 2]. DNA methylation is usually quantified between 0 and 1, and represents the proportion of methylated DNA molecules at the CpG site in the measured tissue. The initial analyses involve univariate testing of each CpG site (e.g. on the Illumina HumanMethylation450 array there are ~485,000 CpG sites [3]) to identify DNA methylation that is associated with an exposure and/or a phenotype [4] while accounting for multiple testing (a Bonferroni corrected *p*-value threshold of $\sim1\times{10}^{-7}$ is often used for studies based on the Illumina HumanMethylation450 array). Methylation at CpG sites is often treated as the outcome (i.e. a possible consequence of the trait) [4], where mean levels of methylation are regressed against the exposure using a linear regression model. These analyses are usually adjusted for batch effects and other technical covariates [5], as well as for cell composition [6] and other potential confounding factors such as age, gender and other characteristics.

## Modelling approaches

### Location tests

Linear regression is commonly used to assess mean differences in methylation by an exposure. That is,

$$y_{i}=\alpha+x_{i}^{'}\beta+\epsilon_{i} , i=1,\ldots,n , (1)$$

where $y_{i}$ is the outcome for the $i$-th individual (usually DNA methylation measurements in EWAS), $x_{i}$ is the exposure(s) for the $i$-th individual and $\epsilon_{i}\sim N(0, \sigma_{\epsilon}^{2})$. Linear regression is known to be relatively robust to the underlying assumptions related to the residuals when estimating the regression coefficients, particularly the estimated regression coefficient related to the exposure(s), i.e. $\hat{\beta}$, as this is unaffected by the residual mean-zero assumption being violated. Moreover, the assumption that the residuals are normally distributed has less impact on the statistical inference of the regression coefficients with increasing sample size, as for large sample sizes ($n$ > ~100) the central limit theorem applies. The main limitation of use of linear regression in the context of analysing DNA methylation data is that it is affected by outlying methylation values. However, robust regression techniques [7] have been developed to handle outlying values in the outcome (e.g. least absolute deviation regression and M-estimators), so this issue is not considered further here.

### Bartlett’s test of variability

Bartlett’s test [8] tests the null hypothesis that all $k$ group variances are the same against the alternative that at least one pair of variances differ. That is, let $y_{ij}$ be the outcome of the $i$-th individual in the $j$-th group and $y_{ij}\sim N\left( \mu_{j},\sigma_{j}^{2} \right)$, where $\mu_{j}$ and $\sigma_{j}^{2}$ are the mean and variance of $y_{ij}$ in the $j$-th group, then the Bartlett’s test statistic for $H_{0}:\sigma_{1}^{2}=\sigma_{2}^{2}=\ldots=\sigma_{k}^{2}$ is given by

$$BT= \frac{\left( n-k \right)\log\left( S_{p}^{2} \right)- \sum_{j=1}^{k} \left( n_{j}-1 \right)\log(S_{j}^{2})}{1+\frac{1}{3(k-1)}\left( \sum_{j=1}^{k} \left( \frac{1}{n_{j}-1} \right)-\frac{1}{n-k} \right)} \sim\chi_{k-1}^{2} , (2)$$

where $n$ is the total number of individuals, $n_{j}$ is the number of individuals in the $j$-th group and $S_{j}^{2}$ and $S_{p}^{2}$ are the group variance and the pooled variance estimates (i.e. $S_{p}^{2}=\frac{1}{n-k}\sum_{j=1}^{k} \left( n_{j}-1 \right)\log\left( S_{j}^{2} \right)$), respectively.

### Brown-Forsythe test of variability

The Brown-Forsythe test [9] is essentially a one-way analysis of variability of the variable $z_{ij}=|y_{ij} -y_{mj}|$ where $y_{ij}$ is the methylation of the $i$-th individual in the $j$-th group and $y_{mj}$ is the median of the $j$-th group. Hence, the Brown-Forsythe test statistic for $H_{0}:\sigma_{1}^{2}=\sigma_{2}^{2}=\ldots=\sigma_{k}^{2}$ is given by

$$BF= \frac{\left( n-k \right)\sum_{j=1}^{k} n_{j}\left( \bar{z}_{j}-\bar{z} \right)^{2}}{(k-1)\sum_{j=1}^{k} \sum_{i=1}^{n_{j}} \left( z_{ij}-\bar{z}_{j} \right)^{2}} \sim F_{k-1,n-k} , (3)$$

where $k$ is the number of groups, $n_{j}$ is the number of individuals in the $j$-th group and $\bar{z}_{j}$ and $\bar{z}$ are the group mean and overall mean of $z_{ij}$, respectively.

### Likelihood ratio tests

Linear mixed models [10] can be used to construct a likelihood ratio test (LRT) of the mean and variability or the variability only. Suppose $y_{i}$ is the response variable for the $i$-th sample and $x_{i}$ is either the binary or continuous exposure, then consider the following model (model 1):

$$y_{i}=\tilde{\alpha}+\tilde{\beta}x_{i}+\epsilon_{i} , i=1,\ldots,n , (4)$$

where

$$\tilde{\alpha}=\alpha_{0}+u_{1i} ,$$

$$\tilde{\beta}=\beta_{0}+u_{2i} (5)$$

and $u_{i} \sim N(0, \Sigma_{u})$ (where $\Sigma_{u}$is an unstructured covariance matrix) and $\epsilon_{i}\sim N(0, \sigma_{e}^{2})$.

Consider also the model (model 0):

$$y_{i}=\tilde{\alpha}+\epsilon_{i} , i=1,\ldots,n . (6)$$

Then to test for a mean and/or variability effect the deviance from model 0 minus the deviance from model 1 is tested against $\chi_{2}^{2}$, i.e. $D\left( model 0 \right)-D\left( model 1 \right) \sim\chi_{2}^{2}$. Likewise, to test for a variability effect only, the deviance from model 1 is tested against the deviance from the same model without including the $u_{2i}$ term using $\chi_{1}^{2}$. For continuous exposures this model assesses the relationship of the variance of $y_{i}$ with $x_{i}$ and $x_{i}^{2}$.

Similarly, a LRT can be constructed using the deviances from double generalized linear models [11] with and without mean and dispersion parameters related to the exposure ($x_{i}$). The difference here is that a log link is used to model the variability. However, for categorical exposures these tests are identical and yield the same results as those from the LRT proposed by Cao *et al*. [12].

### Joint location-and-scale test using Fisher’s method (JLSp)

If the outcome data are symmetrically distributed then the $p$-values from the location and scale tests are independent and can be combined using Fisher’s method (JLSp) [13, 14]. That is, we can combine the $p$-value from the location test in (Eq. 1) ($p_{l}$) with the $p$-value from the location test in (Eq. 3) ($p_{s}$) using the following test statistic:

$$Q=-2\left( \log\left( p_{l} \right)+\log\left( p_{s} \right) \right) \sim\chi_{4}^{2} . (7)$$

### Joint location-and-scale score test (JLSsc)

We propose to test the joint null hypothesis $H_{0}:\beta=\delta=0$ in the model specification:

$$y_{i}=\alpha+x_{i}^{'}\beta+\varepsilon_{i}$$

$$\left( y_{i}-\bar{y} \right)^{2}=\gamma+x_{i}^{'}\delta+u_{i} , (8)$$

where $x_{i}$ is a ($k_{x}$) vector of exposures and $\bar{y}$ is the sample average of $y_{i}$. The first part, $H_{0}:\beta=0$, is the null hypothesis that $x$ does not affect the mean of $y$. The second part, $H_{0}:\delta=0$, is the null hypothesis that $x$ does not affect the variability of $y$. The second equation is essentially the Breusch-Pagan test, except that the variance is calculated under the null that $\beta=0$.

Let $\tilde{y}_{i}=y_{i}-\bar{y}$, $\tilde{x}_{i}=x_{i}-\bar{x}$ and $\tilde{d}_{i}=\tilde{y}_{i}^{2}-\hat{\sigma}^{2}$, where $\hat{\sigma}^{2}=\frac{1}{n}\sum_{i=1}^{n} \tilde{y}_{i}^{2}$. Further, let the $n\times k_{x}$ matrix $\tilde{X}=\left[ \tilde{x}_{i}^{'} \right]$ and the $n$ vectors $\tilde{y}=\left( \tilde{y}_{i} \right)$ and $\tilde{d}=\left( \tilde{d}_{i} \right)$. Then the linear regression estimators for $\beta$ and $\delta$ are given by

$$\hat{\beta}=\left( \tilde{X}^{'}\tilde{X} \right)^{-1}\tilde{X}^{'}\tilde{y}$$

$$\hat{\delta}=\left( \tilde{X}^{'}\tilde{X} \right)^{-1}\tilde{X}^{'}\tilde{d} . (9)$$

Let $\theta=\left( \begin{matrix} \beta\\ \delta\end{matrix} \right), \hat{\theta}=\left( \begin{matrix} \hat{\beta} \\ \hat{\delta} \end{matrix} \right)$ and $\hat{\Sigma}=\frac{1}{n}\sum_{i=1}^{n} \left[ \begin{matrix} \tilde{y}_{i}^{2} & \tilde{y}_{i}\tilde{d}_{i} \\ \tilde{y}_{i}\tilde{d}_{i} & \tilde{d}_{i}^{2} \end{matrix} \right]$. The estimator for the variance of $\hat{\theta}$ under the null that $\beta=\delta=0$ and the additional assumption that the conditional skewness and kurtosis of $y_{i}$ do not vary with the values of $x_{i}$, is then given by

$$V\hat{a}r\left( \hat{\theta} \right)={\hat{\Sigma}\otimes\left( \tilde{X}^{'}\tilde{X} \right)}^{-1} . (10)$$

A test statistic for testing for $H_{0}:\beta=\delta=0$, or $H_{0}:\theta=0,$ is then given by

$$S=\hat{\theta}^{'}\left( \hat{\Sigma}^{-1}\otimes\left( \tilde{X}^{'}\tilde{X} \right) \right)\hat{\theta} . (11)$$

It follows from standard limiting distribution theory that, under the null, $S\underset{\to}{d}\chi_{2k_{x}}^{2}$. The restrictions on conditional skewness and kurtosis are needed for the simple Kronecker version of the variance estimator as given in (Eq. 10) to be valid, as $\tilde{y}_{i}\tilde{d}_{i}$ and $\tilde{d}_{i}^{2}$ contain third and fourth order moments. This version of the test was used in the simulations and applied examples unless stated otherwise. The proposed test using statistic *S* (Eq. 11) is a score test based on the joint asymptotic distribution of $\hat{\beta}$ and $\hat{\delta}$.

To see the relation between the score test and estimating equations more clearly, consider the random variables $Y$ and $X$, with the conditional distribution of $Y$ given $X$ given by

$$Y|X=x\sim N\left( \alpha+x^{'}\beta,\sigma^{2}\left( x \right) \right) . (12)$$

We would like to test the joint hypothesis $H_{0}:\beta=0,\sigma^{2}\left( x \right)=\sigma^{2}$, i.e. the joint null hypothesis that $\beta=0$ and that the conditional variance is not a function of $x$, also called homoskedasticity.

A standard test for homoskedasticity was proposed by Breusch and Pagan. For the general specification $\sigma^{2}\left( x \right)=h\left( \gamma+x^{'}\delta\right)$, with $h\left( . \right)$ a strictly positive function, the score test statistic for the null $H_{0}:\delta=0$ can be obtained for the sample $\left\{ y_{i},x_{i}^{'} \right\}_{i=1}^{n}$ as $nR^{2}$ in the linear regression of $\hat{\varepsilon}_{i}^{2}$ on a constant and $x_{i}$,

$$\hat{\varepsilon}_{i}^{2}=\gamma+x_{i}^{'}\delta+v_{i} (13)$$

where $\hat{\varepsilon}_{i}=y_{i}-x_{i}^{'}\hat{\beta}$, and $\hat{\beta}$ is the ordinary least squares (OLS) estimator in the linear model

$$y_{i}=\alpha+x_{i}^{'}\beta+\varepsilon_{i} , (14)$$

which is the ML estimator under the null that $\delta=0.$ Note that the $nR^{2}$ statistic is equivalent to the score test statistic in model (Eq. 1) assuming normality, but this is not an assumption needed, just an equivalence result.

Let $\hat{\sigma}^{2}=\frac{1}{n}\sum_{i=1}^{n} \hat{\varepsilon}_{i}^{2}$, then the $nR^{2}$ version of the Breusch-Pagan test statistic is given by

$$BP=nR^{2}=\frac{\left( \sum_{i=1}^{n} \left( \hat{\varepsilon}_{i}^{2}-\hat{\sigma}^{2} \right)z_{i} \right)^{'}\left( \sum_{i=1}^{n} z_{i}z_{i}^{'} \right)^{-1}\left( \sum_{i=1}^{n} \left( \hat{\varepsilon}_{i}^{2}-\hat{\sigma}^{2} \right)z_{i} \right)}{\frac{1}{n}\sum_{i=1}^{n} \left( \hat{\varepsilon}_{i}^{2}-\hat{\sigma}^{2} \right)^{2}} (15)$$

where $z_{i}=\left( 1,x_{i}^{'} \right)^{'}$.

For our joint test, for the homoskedasticity part, we impose that $\beta=0$ and hence

$$\hat{\varepsilon}_{i,\left( \beta=0 \right)}^{2}=\left( y_{i}-\bar{y} \right)^{2} , (16)$$

where $\bar{y}=\frac{1}{n}\sum_{i=1}^{n} y_{i}$, and we operationalise the test for the joint null by combining the linear model with the Breusch-Pagan equation

$$y_{i}=\alpha+x_{i}^{'}\beta+\varepsilon_{i}$$

$$\left( y_{i}-\bar{y} \right)^{2}=\gamma+x_{i}^{'}\delta+u_{i} (17)$$

Note that under normality, under the null, skewness and kurtosis are clearly not a function of $x$.

In terms of method of moments, let

$$g_{i}=\left( \begin{matrix} \tilde{x}_{i}\tilde{y}_{i} \\ \tilde{x}_{i}\tilde{d}_{i} \end{matrix} \right)=\left( I_{2}\otimes\tilde{x}_{i} \right)\left( \begin{matrix} \tilde{y}_{i} \\ \tilde{d}_{i} \end{matrix} \right) (18)$$

and

$$\hat{V}=\hat{\Sigma}\otimes\left( \frac{\tilde{X}^{'}\tilde{X}}{n} \right) (19)$$

Then, with $\bar{g}=\frac{1}{n}\sum_{i=1}^{n} g_{i}$, it follows that

$$S=n\bar{g}^{'}\hat{V}^{-1}\bar{g}\underset{\to}{d}\chi_{2k_{x}}^{2} . (20)$$

Alternatively, relaxing the constant skewness and kurtosis assumption, a robust version of the test is given by

$$S_{r}= n\bar{g}^{'}\hat{V}_{r}^{-1}\bar{g}\underset{\to}{d}\chi_{2k_{x}}^{2} , (21)$$

where $\hat{V}_{r}=\frac{1}{n}\sum_{i=1}^{n} g_{i}g_{i}^{'}$.

#### Brown-Forsythe methodology

JLSsc can also be set-up using the Brown-Forsythe methodology by replacing $\tilde{d}_{i}=\tilde{y}_{i}^{2}-\hat{\sigma}^{2}$ with $\tilde{d}_{i}=|y_{i}-y|- \frac{1}{n}\sum_{i=1}^{n} |y_{i}-y|$ in equations (Eqs. 9-11 & 18-21) where $y$ is the median of $y$.

#### Conditioning on additional covariates

Additional terms such as the square of a continuous exposure, especially useful for modelling the relationship with outcome variability, can be added as part of $x_{i}$ vector and would be included in both parts of the test. Other variables that are expected to affect the outcome but are not considered important for testing purposes are regressed out of both the outcome and exposure variables by taking residuals from linear regression adjusting for these variables prior to analysis with JLSsc. Suppose we have a variable $W$ that we wish to remove from the exposure and outcome before testing – i.e. we think that $W$ may cause outcome, variability in outcome, or both – but there is no interest in testing this. As with usual confounding, the interest is in examining the effect of $X$ on mean and variability of $Y$, conditioned on $W$. One approach would be to adjust for $W$ in both regressions:

$$Y=\alpha+X^{'}\beta+\gamma W+\varepsilon$$

$$\sigma^{2}=\lambda+X^{'}\delta+\tau W+u (22)$$

Instead, we use the results of the Frisch-Waugh-Lovell theorem, which states that the least squares regression of $Y$ on $X$ and $W$ will give exactly the same coefficient for $X$, and residuals, as the least squares regression of ($Y$ adjusted for $W$) on ($X$ adjusted for $W$). So, first we partial out $W$ from $Y$ and $X$:

$$Y=A+BW+E$$

$$X=a+Wb+e (23)$$

Then we use the sample residuals, i.e. the estimates of $E$ and $e$, $\hat{E_{i}}$ and $\hat{e}_{i}$ in place of $y_{i}$ and $x_{i}$ in the test procedure. This is exactly equivalent to the procedure above, adjusting for $W$ at all stages, but can be computationally easier if $W$ is high-dimensional.

## Simulation study

1) Type I error simulation using a methylation dataset (Figs. S2-S8, S11-14 a-b & S18 a-b)

The type I error simulations were performed by randomly generating a binary or continuous exposure and testing the association of this exposure with mean and variability of DNA methylation at each CpG site in Tsaprouni *et al*. [15] dataset (data accessible at NCBI GEO database [16], accession GSE50660). Although the distribution of DNA methylation at some CpG sites is highly skewed or has very thick tails, most have skewness between -1 and 1 (67.4%) and kurtosis less than 3 (74%). Histograms of the mean, standard deviations, skewness and kurtosis of all CpG sites are shown in Fig. S1.

To generate datasets with varying sample size (100, 500, 1000 and 10,000 samples), samples were randomly taken with replacement from the Tsaprouni *et al.* dataset, while adding a small amount of noise by drawing from a random normal distribution with mean zero and the standard deviation set to ten percent of the standard deviation of methylation at that CpG site. The binary and continuous exposures were randomly generated using $Ber(0.5)$ and $N(0,1)$, respectively (Figs. S2-S4 & S8). For approaches which failed to adequately control type I error rates, we repeated the tests after applying M-value (i.e. $\log_{2} (y_{i}/(1-y_{i}))$) and inverse normal rank transformations to the methylation levels (Figs. S5-S7). Additional normally distributed simulations were performed using a categorical exposure ($Bin(2,0.3)$) with three categories (Fig. S11). We also conducted simulations adding a squared exposure term to the JLSsc approach with a continuous exposure (i.e. the simulations remained unchanged, but the JLSsc model included a term for the exposure and for the square of the exposure) (Fig. S12). Further simulations were performed using the normally distributed residual simulation set for the binary and continuous exposures where a single outlier was simulated to be 5 standard deviations away from $\mu$ (Fig. S13).

The Brown-Forsythe formulation of JLSsc was compared to the standard version using the simulated dataset with a binary covariate (simulated as above, Fig. S14).

2) Power simulation using a methylation dataset (Figs. S9-S10, S11-14 c-d & S18 c-d)

Power simulations were performed using the same dataset as above. The power simulations were performed using the following model,

$$y_{i}=\alpha+\beta x_{i}+v_{i}+e_{i} ,$$

where $y_{i}$ is the methylation at the randomly selected CpG site for the $i$-th sample and $x_{i}$ is either the binary or continuous exposure ($x_{i} \sim Ber(0.5)$or $x_{i} \sim N(0,1)$). $\alpha$ is the mean methylation from Tsaprouni *et al.* at the randomly selected CpG site ($\mu$), $\beta=\pm w\sigma$ and $v_{i}\sim N(0,zx_{i}\sigma^{2})$, where $\sigma$ is the standard deviation (SD) of methylation from Tsaprouni *et al.* at the CpG site, $w$ is the scaler of the SD for the mean effect and $z$ is the scaler for the variability effect. The direction of $w$ was set to be positive if $\mu\leq0.5$ and negative if $\mu>0.5$. The residual ($e_{i}$) was set to be either normally distributed [$e_{i} \sim N(0,\sigma^{2})$; $w$= (0, 0.1, …, 0.5) and $z$= (0, 0.05, …, 1) for binary exposures and $w$= (0, 0.025, …, 0.3) and $z$= (0, 0.05, …, 0.5) for continuous exposures] (Fig. 2), heavy-tailed (Fig. S9) [$e_{i} \sim\sigma t_{4}$; $w$= (0, 0.1, …, 0.5) and $z$= (0, 0.05, …, 1) for binary exposures and $e_{i} \sim\sigma t_{8}$; $w$= (0, 0.05, …, 0.5) and $z$= (0, 0.1, …, 1) for continuous exposures] or skewed (Fig. S10) [$e_{i} \sim\frac{\sigma}{2}\chi_{1}^{2}$; $w$= (0, 0.025, …, 0.25) and $z$= (0, 0.05, …, 1) for binary exposures and $w$= (0, 0.025, …, 0.3) and $z$= (0, 0.05, …, 0.5) for continuous exposures]. Additional normally distributed simulations (Fig. S11) were performed using a categorical exposure ($B$in(2,0.3)) with three categories [$w$= (0, 0.1, …, 0.5) and $z$= (0, 0.05, …, 1) for the second category and $w$= (0, 0.2, …, 1) and $z$= (0, 0.2, …, 4) for the third category]. We also conducted simulations adding a squared exposure term to the JLSsc approach with a continuous exposure (i.e. the simulations remained unchanged, but the JLSsc model included a term for the exposure and for the square of the exposure) (Fig. S12). Further simulations were performed using the normally distributed residual simulation set for the binary and continuous exposures where a single outlier was simulated to be 5 standard deviations away from $\mu$ (Fig. S13).

The Brown-Forsythe formulation of JLSsc was compared to the standard version using the simulated dataset with a binary covariate (simulated as above, Fig. S14).

3) Type I error simulation varying the distributions of the outcome variable

We set up further simulations to investigate type I error distributions, drawing the outcome variable distribution from a Normal (0,1), a t-distribution with 4 degrees of freedom, log-normal (0,1) distribution or contaminated Normal 90% N(0,1) & 10% N(5,1), and the exposure as a binary (Fig. S15), three-category (Fig. S16) or a standard Normal variable (Fig. S17). The aim was to estimate the type I error of linear regression (which only tests for a location effect), the Brown-Forsythe test (which tests for a scale effect), JLSp (joint test of location and scale assuming independence of p-values for location and scale) and JLSsc (joint test of location and scale). We carried out 1000 repetitions, with the sample size in each case set to 200. In all simulations, the exposure was independent of the outcome.

4) Type I error and power simulation examining robustness to skew and kurtosis

We set up further simulations to investigate type I error distributions and power, when the assumption of constant skew and kurtosis across exposure levels was not satisfied. The aim was to estimate the type I error of JLSsc (joint test of location and scale) and “robust JLSsc” (joint test not assuming constant skew and kurtosis across levels of exposure). We carried out 1000 repetitions, with the sample size in each case set to 10,000. First, we simulated a continuous exposure independent of the outcome (to examine type I error when the skew and kurtosis assumptions are satisfied, Fig. S18 a,b), and then a continuous exposure with a mean and then a variance effect, but constant skew and kurtosis (to examine power when the skew and kurtosis assumptions are satisfied, Fig. S18 c,d). We then simulated a binary exposure with no mean or variability effect, but an effect on kurtosis (Fig. S19 a,c) and both skew and kurtosis (Fig. S19 b,d) (to examine type I error when the skew and kurtosis assumptions are not satisfied).

## Application to offspring gender and gestational age on cord blood DNA methylation

### Quality control and pre-processing procedures in ARIES

Cord blood samples were collected according to standard procedures. The DNA methylation wet laboratory and pre-processing analyses were performed at the University of Bristol as part of the ARIES project. Following extraction, DNA was bisulphite converted using the Zymo EZ DNA MethylationTM kit (Zymo, Irvine, CA, USA). Following conversion, genome-wide methylation of over 485,000 CpG sites were measured using the Infinium HumanMethylation450 BeadChip according to the standard protocol. The arrays were scanned using an Illumina iScan and initial quality review was assessed using GenomeStudio (version 2011.1).

Samples from all time points in ARIES were distributed across slides using a semi-random approach (sampling criteria were in place to ensure that all time points were represented on each array) to minimize the possibility of confounding by batch effects. In addition, during the data generation process a wide range of batch variables were recorded in a purpose-built laboratory information management system (LIMS). The main batch variable was found to be the bisulphite conversion plate number. Samples were converted in batches of 48 samples and each batch identified by a plate number. The LIMS also reported quality control (QC) metrics from the standard control probes on the 450K BeadChip for each sample. Samples with more than 5% of probes that have detection $p>$ 0.01 were excluded from the analysis. As an additional QC step genotype probes were compared with SNP-chip data from the same individual to identify and remove any sample mismatches. For individuals with no genome-wide SNP data, samples were flagged if there was a sex-mismatch based on X and Y chromosome methylation.

In addition to these QC steps, probes that had detection $p>$ 0.01 for more than 5% of samples were excluded from analysis. After excluding these probes as well as probes on the X or Y chromosomes, a total of 468,611 CpG sites were included in the main analysis. Raw probe intensities were normalized using functional normalization with the meffil package [17, 18]. Methylation levels at each CpG site 5 standard deviations away from the mean were excluded.

### Gestational age

Gestational age was calculated (in weeks) based on the date of the mother’s last menstrual period when the mother was certain of this, but for uncertain last menstrual periods and conflicts with clinical assessment the ultrasound assessment was used. Where maternal report and ultrasound assessment conflicted, an experienced obstetrician reviewed clinical records and made a best estimate.

### Results

Methylation at 8,174 CpG sites were associated with gender in cord blood (through the mean, variability or joint tests; Fig. 3a and Table S3). Most of these sites were identified through a mean difference in methylation of males and females (7,642 CpGs had a mean difference with $p<1\times{10}^{-7}$). 240 CpG sites were associated with a variability difference between males and females, of which all but 12 were also associated with a mean difference. For instance, cg18918831 was more variable in males compared to females (Fig. S22). The joint location-and-scale tests identified 7,724 of these CpG sites (JLSp identified 7,213 sites and JLSsc identified 7,228 sites), including all of those with a variability effect (Table S3). Using only the mean (regression) and variability (Brown-Forsythe) tests separately (taking account of the increasing number of tests done by using the cut-off $p<5\times{10}^{-8}$ ) would have identified 7,244 as having either a mean or a variability difference (or both). Of these, 6,967 (96%) and 6,744 (93%) were identified by JLSsc and JLSp respectively. The joint tests additionally identified 673 sites not identified by the mean or variability tests alone (204 sites with JLSsc only, 412 sites with JLSp only and 57 sites with both).

Gestational age was associated with cord blood methylation at 412 CpG sites (Fig. 3b and Table S4). Most of these CpG sites (354, 86%) were associated with a mean effect of gestational age on methylation, and there were no CpG sites with a variability effect with $p<1\times{10}^{-7}$. The joint mean and variability tests identified 93.7% of the CpG site associations (JLSp identified 317 and JLSsc identified 340 CpG sites, respectively), including sites that were mostly identified through a variability association (e.g. cg24577594; Table S4). Using only the mean (regression) and variability (Brown-Forsythe) tests separately (taking account of the increasing number of tests done by using the cut-off $p<5\times{10}^{-8}$ ) would have identified 319 CpG sites as having either a mean or a variability difference (or both), all of which had a mean effect. Of these, 311 (97%) and 263 (82%) were identified by JLSsc and JLSp respectively. The joint tests additionally identified 73 sites not identified by the mean or variability tests alone (19 sites with JLSsc only, 44 sites with JLSp only and 10 sites with both).

## References

1. Rakyan VK, Down TA, Balding DJ, Beck S. Epigenome-wide association studies for common human diseases. Nat Rev Genet. 2011;12(8):529-41. doi:10.1038/nrg3000.

2. Flanagan JM. Epigenome-wide association studies (EWAS): past, present, and future. Methods Mol Biol. 2015;1238:51-63. doi:10.1007/978-1-4939-1804-1_3.

3. Dedeurwaerder S, Defrance M, Calonne E, Denis H, Sotiriou C, Fuks F. Evaluation of the Infinium Methylation 450K technology. Epigenomics. 2011;3(6):771-84. doi:10.2217/epi.11.105.

4. Michels KB, Binder AM, Dedeurwaerder S, Epstein CB, Greally JM, Gut I et al. Recommendations for the design and analysis of epigenome-wide association studies. Nat Methods. 2013;10(10):949-55. doi:10.1038/nmeth.2632.

5. Johnson WE, Li C, Rabinovic A. Adjusting batch effects in microarray expression data using empirical Bayes methods. Biostatistics. 2007;8(1):118-27. doi:10.1093/biostatistics/kxj037.

6. Houseman EA, Molitor J, Marsit CJ. Reference-free cell mixture adjustments in analysis of DNA methylation data. Bioinformatics. 2014;30(10):1431-9. doi:10.1093/bioinformatics/btu029.

7. Rousseeuw PJ, Leroy AM. Robust regression and outlier detection. Wiley series in probability and mathematical statistics Applied probability and statistics,. New York: Wiley; 1987.

8. Bartlett MS. Properties of sufficiency and statistical tests. Proceedings of the Royal Society of London Series a-Mathematical and Physical Sciences. 1937;160(A901):0268-82. doi:DOI 10.1098/rspa.1937.0109.

9. Brown MB, Forsythe AB. Robust Tests for Equality of Variances. Journal of the American Statistical Association. 1974;69(346):364-7. doi:Doi 10.2307/2285659.

10. Goldstein H. Multilevel statistical models. 4th ed. Wiley series in probability and statistics. Chichester, West Sussex: Wiley; 2011.

11. Smyth GK. Generalized Linear-Models with Varying Dispersion. Journal of the Royal Statistical Society Series B-Methodological. 1989;51(1):47-60.

12. Cao Y, Wei P, Bailey M, Kauwe JSK, Maxwell TJ. A versatile omnibus test for detecting mean and variance heterogeneity. Genet Epidemiol. 2014;38(1):51-9.

13. Soave D, Corvol H, Panjwani N, Gong J, Li W, Boelle PY et al. A Joint Location-Scale Test Improves Power to Detect Associated SNPs, Gene Sets, and Pathways. Am J Hum Genet. 2015;97(1):125-38. doi:10.1016/j.ajhg.2015.05.015.

14. Soave D, Sun L. A generalized Levene's scale test for variance heterogeneity in the presence of sample correlation and group uncertainty. Biometrics. 2017;73(3):960-71. doi:10.1111/biom.12651.

15. Tsaprouni LG, Yang TP, Bell J, Dick KJ, Kanoni S, Nisbet J et al. Cigarette smoking reduces DNA methylation levels at multiple genomic loci but the effect is partially reversible upon cessation. Epigenetics. 2014;9(10):1382-96. doi:10.4161/15592294.2014.969637.

16. Edgar R, Domrachev M, Lash AE. Gene Expression Omnibus: NCBI gene expression and hybridization array data repository. Nucleic Acids Res. 2002;30(1):207-10.

17. Fortin JP, Labbe A, Lemire M, Zanke BW, Hudson TJ, Fertig EJ et al. Functional normalization of 450k methylation array data improves replication in large cancer studies. Genome Biol. 2014;15(12):503. doi:10.1186/s13059-014-0503-2.

18. Min J, Hemani G, Davey Smith G, Relton CL, Suderman M. Meffil: efficient normalisation and analysis of very large DNA methylation samples. bioRxiv. 2017.

# Supplementary Table Legends

Table S1: Type I error simulations for a standard normal distribution, a t-distribution (4 degrees of freedom), log-normal distribution and a contaminated normal distribution.

Table S2: Characteristics of the mother-offspring pairs with complete covariate information in ARIES.

Table S3: Epigenome-wide association results for gender in cord blood methylation.

Table S4: Epigenome-wide association results for gestational age in cord blood methylation.

# Supplementary Figures

Fig. S1: Histograms of the mean, standard deviation (sd), skewness and kurtosis of the CpG sites in the Tsaprouni *et al.* dataset.


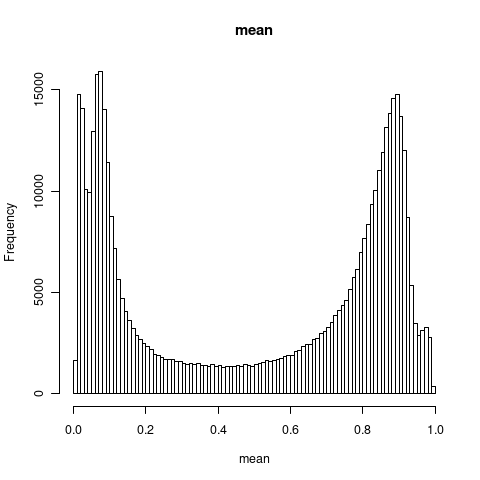

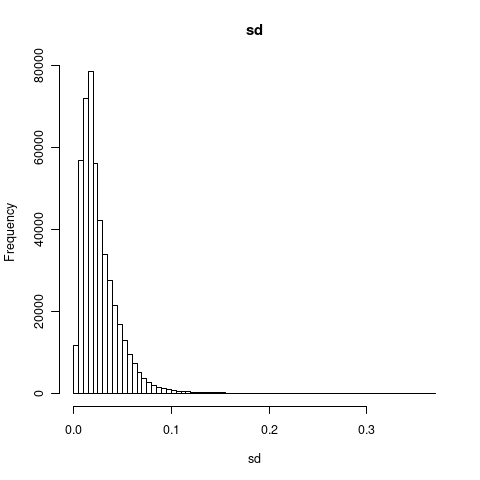


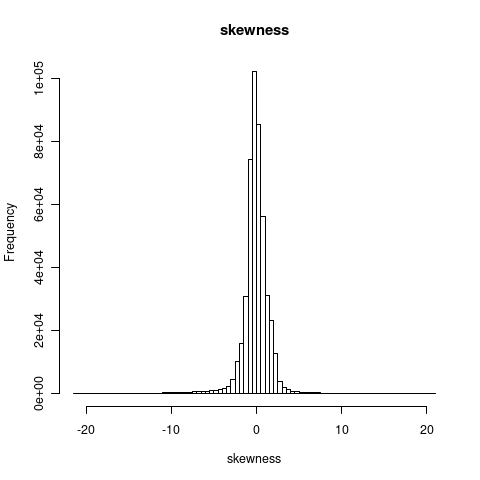

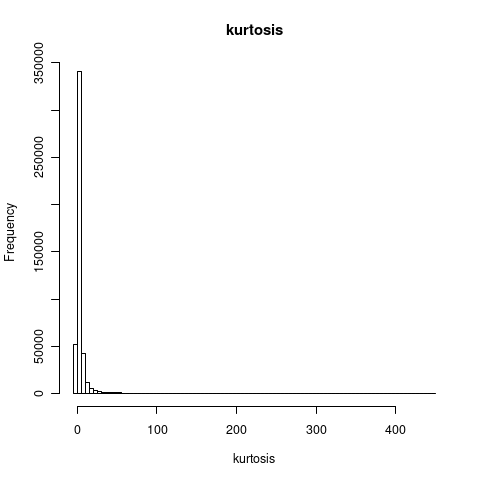


Fig. S2: QQ plots for linear regression (mean test) in type I error simulations. a) binary exposure in 100 samples. b) binary exposure in 500 samples. c) continuous exposure in 100 samples. d) continuous exposure in 500 samples.


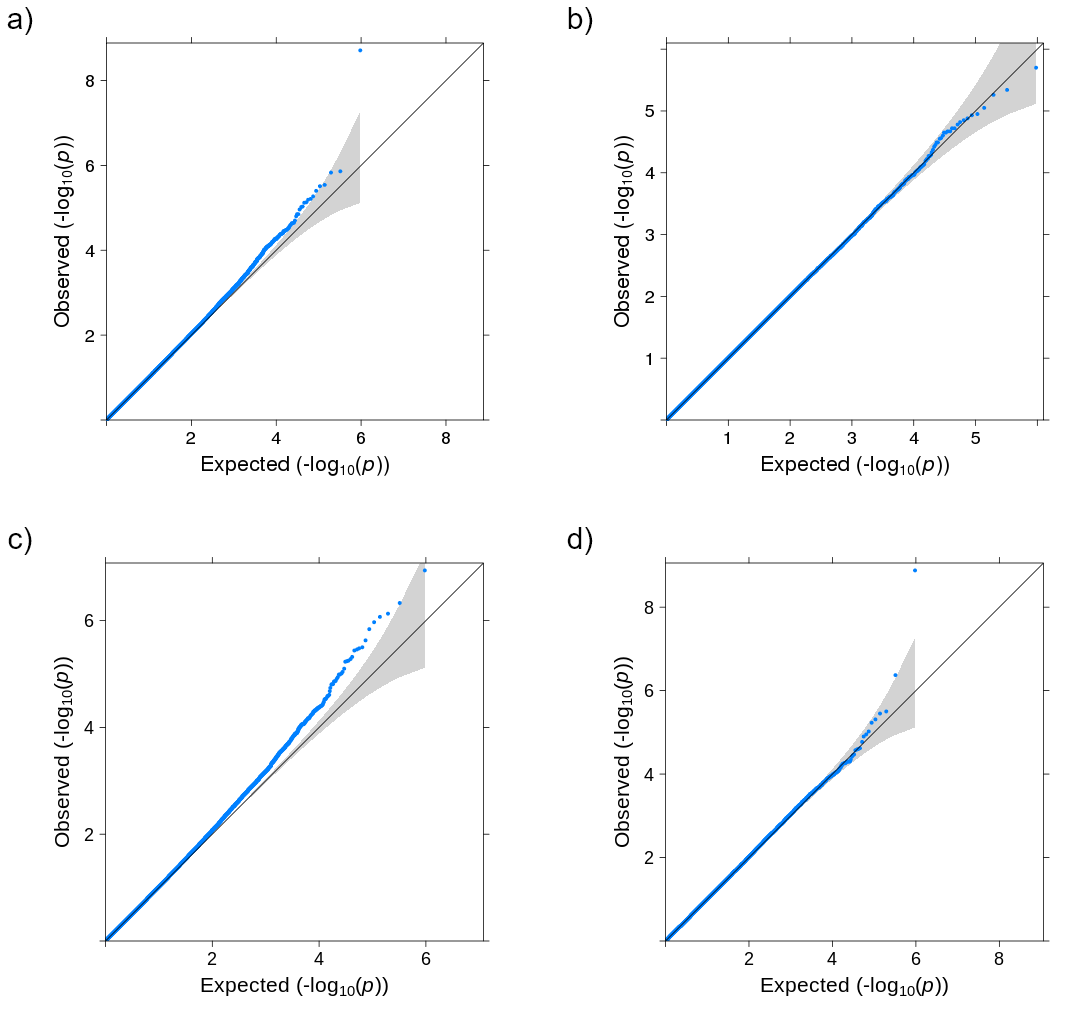


Fig. S3: QQ plots for Brown-Forsythe test (variability test) in type I error simulations. a) binary exposure in 100 samples. b) binary exposure in 500 samples. c) continuous exposure in 100 samples. d) continuous exposure in 500 samples.


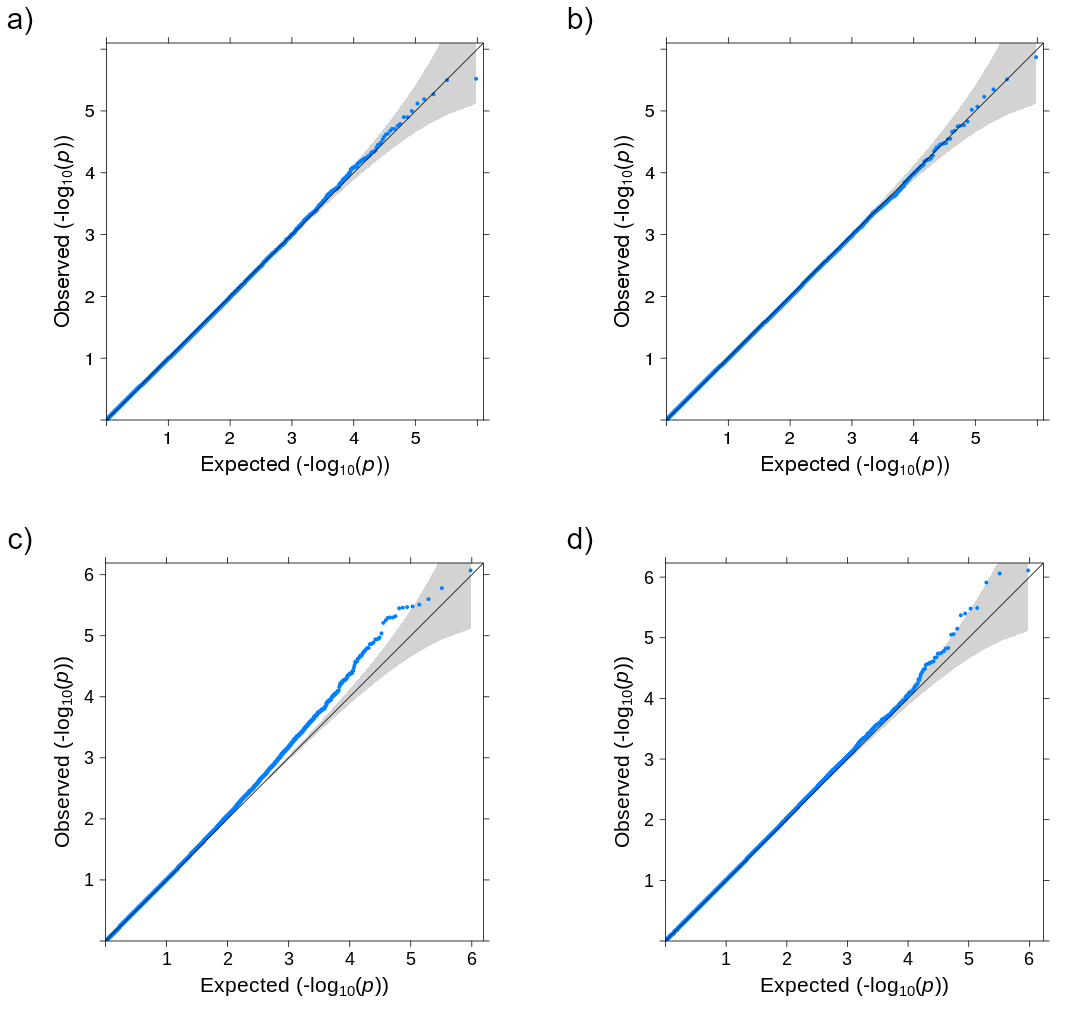


Fig. S4: QQ plots for type I error simulations for a binary exposure in 1000 samples. a) Bartlett’s test (variability test). b) LRTv (variability test). c) LRTmv (joint test). d) DGLM (joint test).


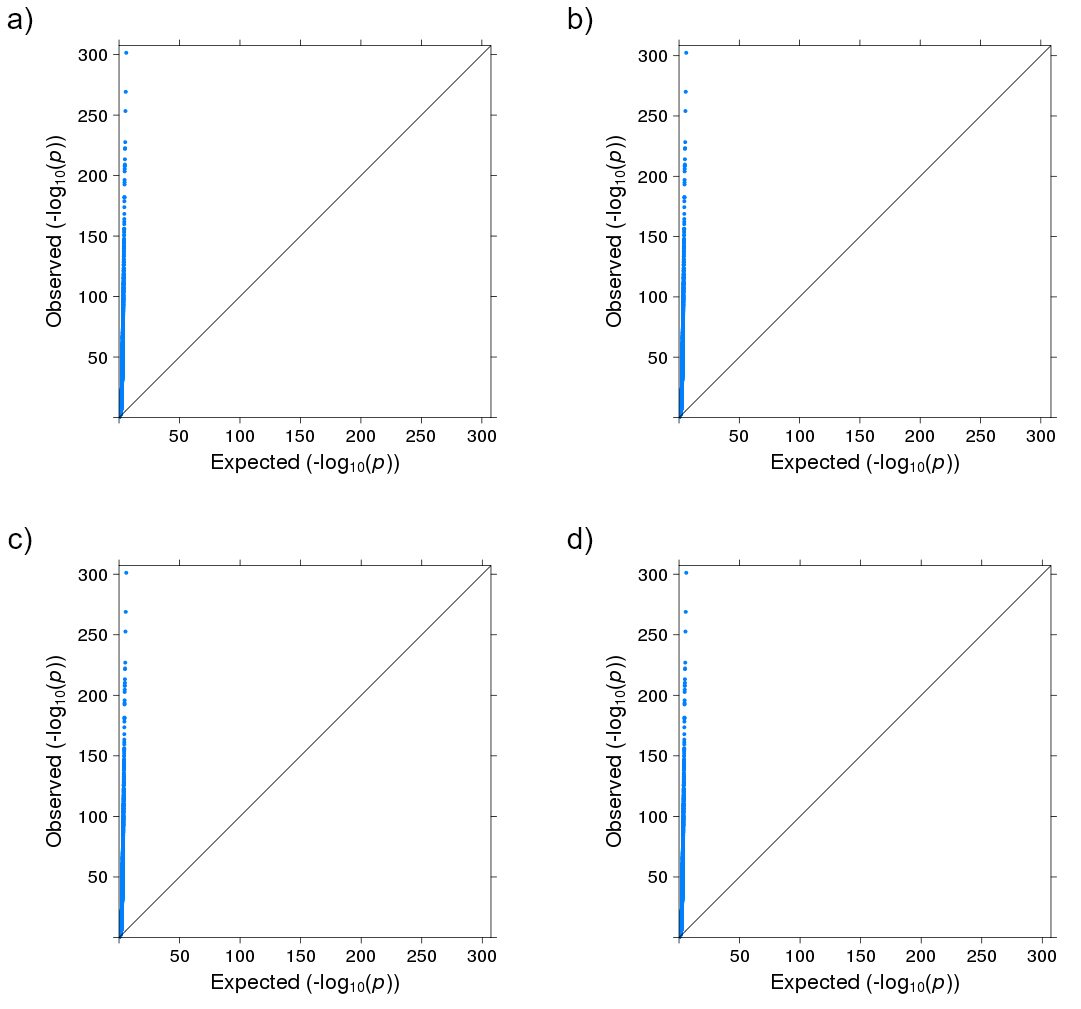


Fig. S5: QQ plots for type I error simulations for a binary exposure in 1000 samples after transforming methylation levels using the M-value transformation. a) Bartlett’s test (variability test). b) LRTv (variability test). c) LRTmv (joint test). d) DGLM (joint test).


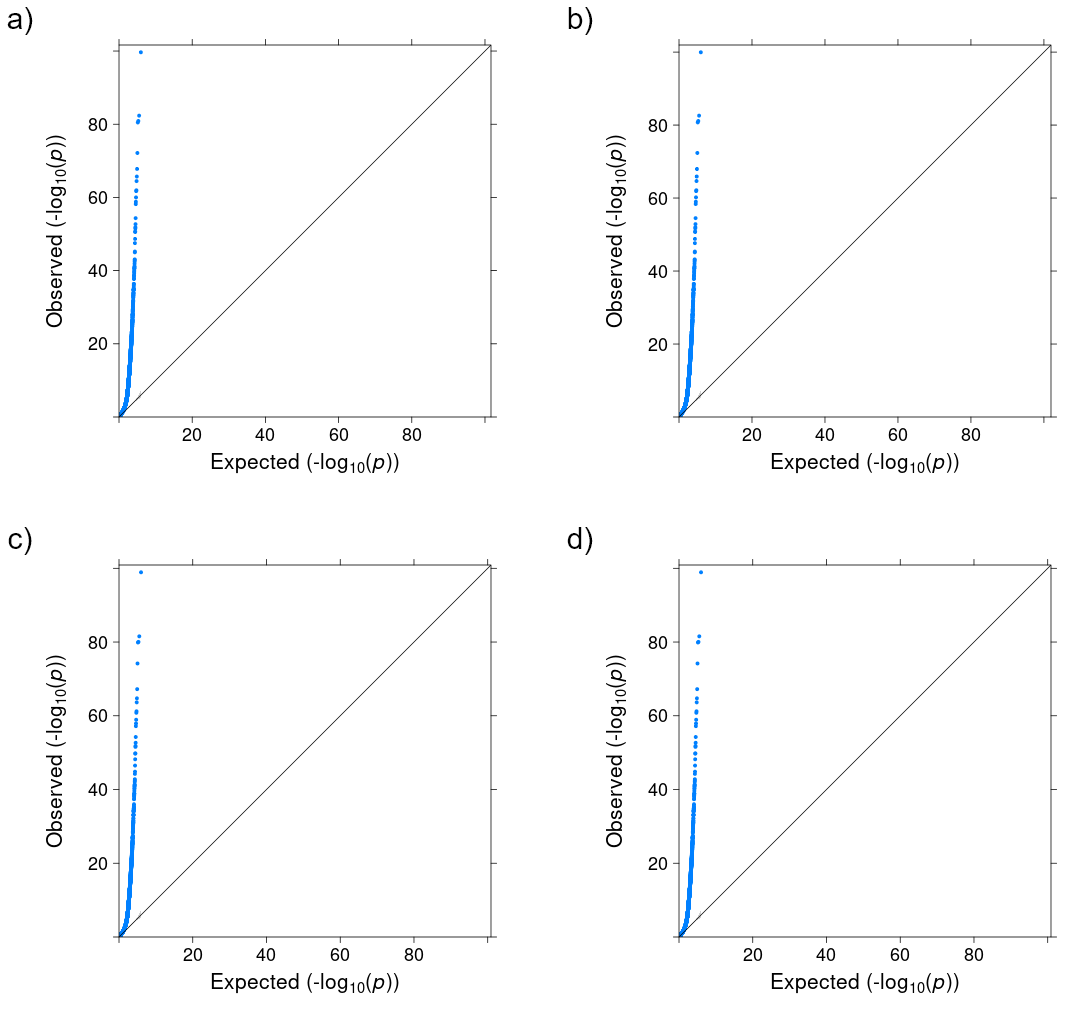


Fig. S6: QQ plots for type I error simulations for a binary exposure in 1000 samples after transforming methylation levels using an inverse normal rank transformation. a) Bartlett’s test (variability test). b) LRTv (variability test). c) LRTmv (joint test). d) DGLM (joint test).


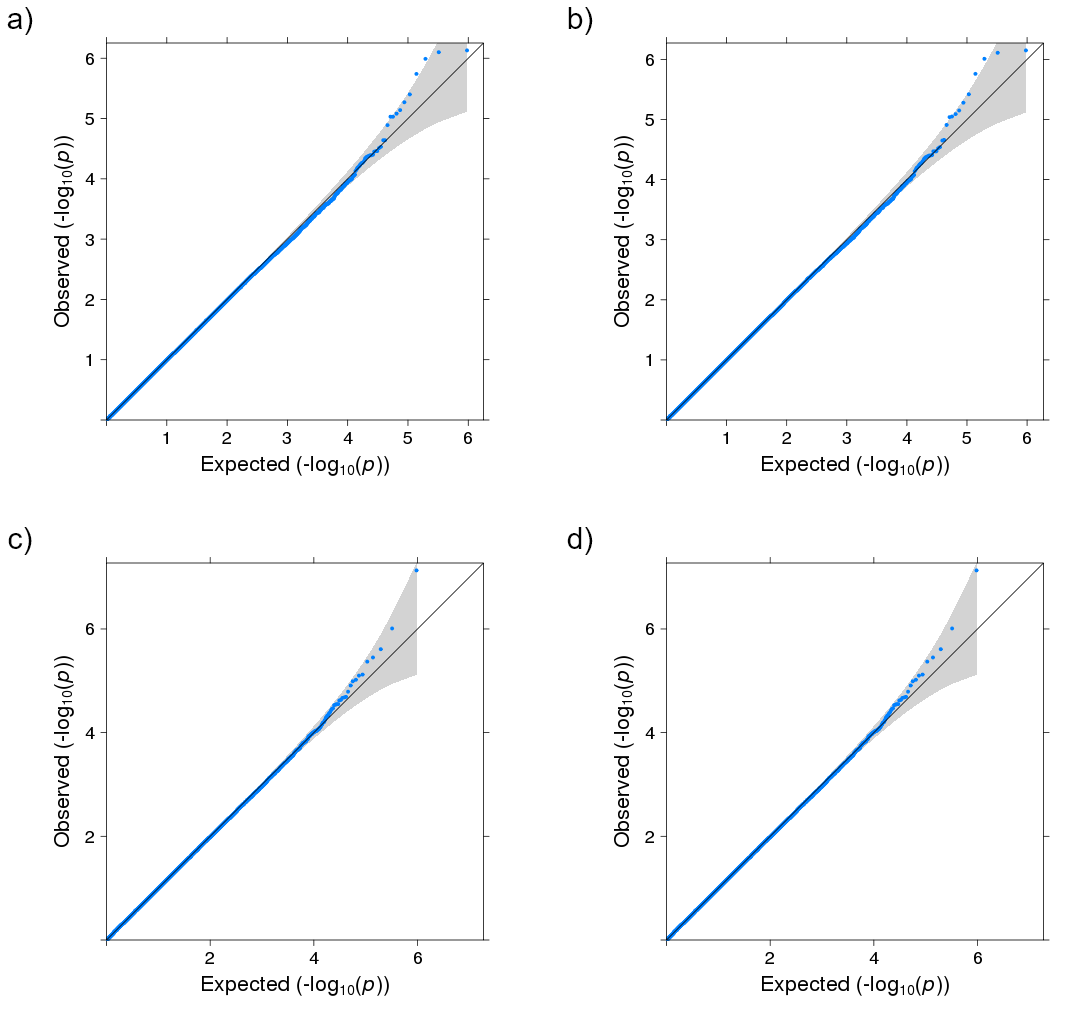


Fig. S7: QQ plots for type I error simulations for a binary exposure in 1000 samples after transforming the methylation levels using an inverse normal rank transformation where the residual error is skewed. a) OLS regression (mean test) where there is a variability effect of 0.5 but no mean effect. b) Brown-Forsythe test (variability test) where there is a mean effect of 0.1 but no variance effect.


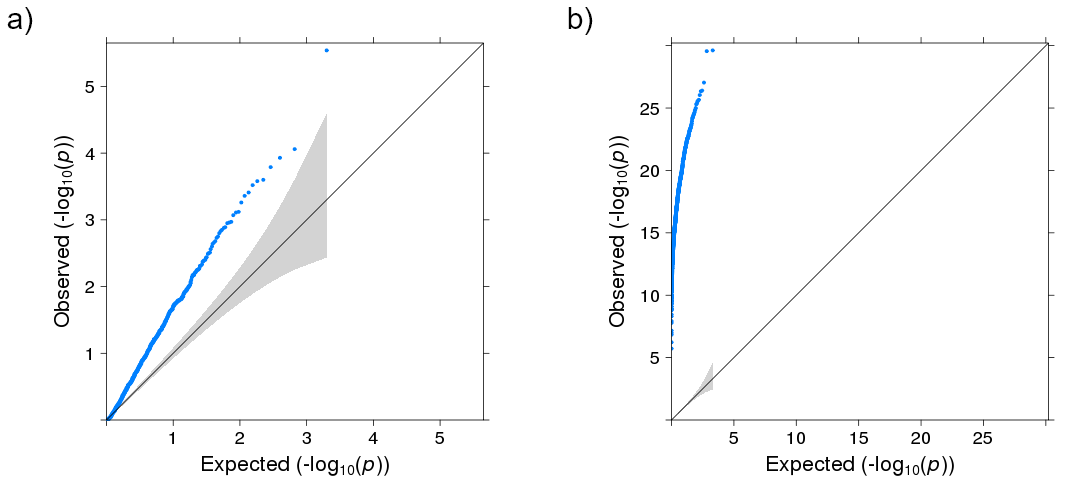


Fig. S8: QQ plots for JLSsc (joint test) and JLSp (joint test) in type I error simulations using 1000 samples. a) JLSsc for a binary exposure. b) JLSsc for a continuous exposure. c) JLSp for a binary exposure. d) JLSp for a continuous exposure.


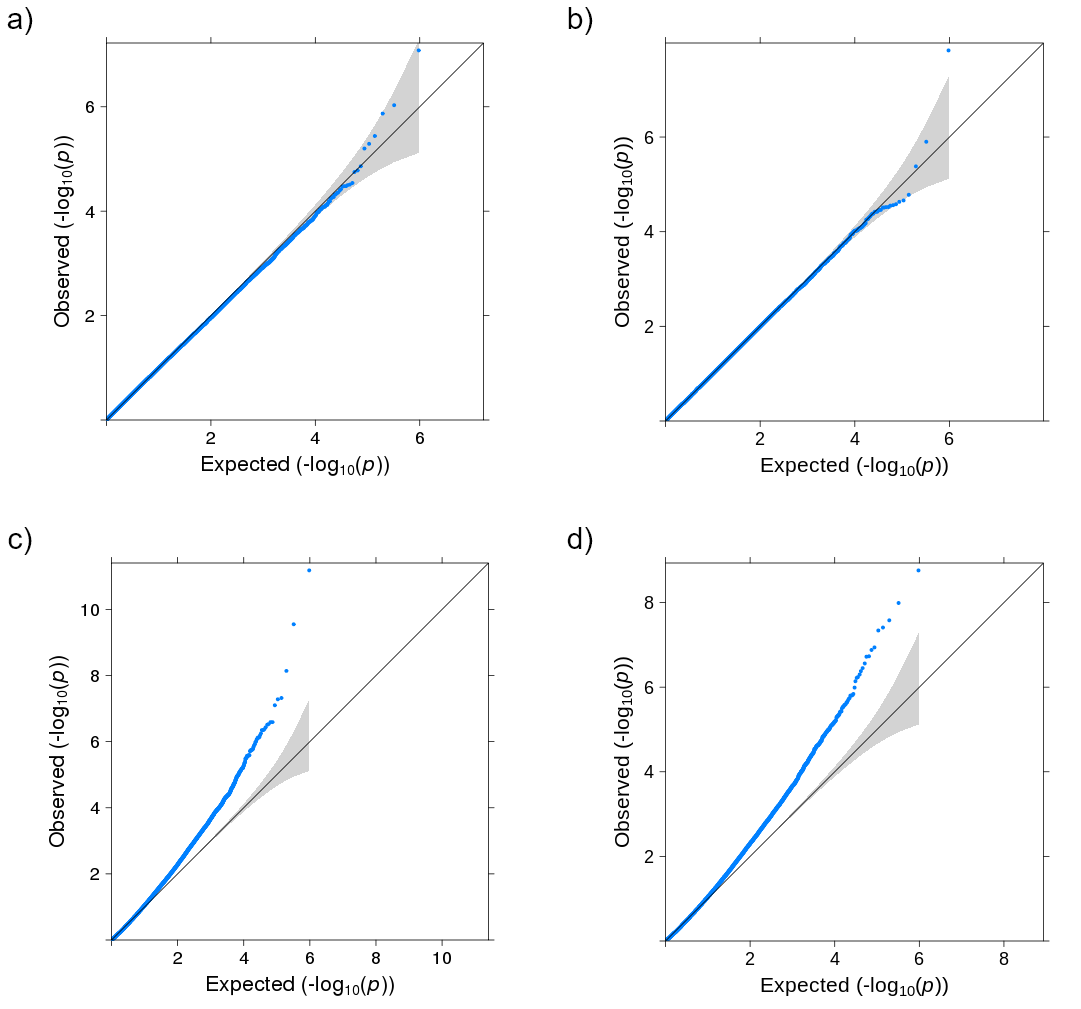


Fig. S9: Power simulation results from a heavy-tailed distribution comparing approaches for identifying CpG sites associated with either a mean and/or a variance effect with the exposure at $p<1\times{10}^{-7}$. a) & b) are plots for a binary exposure and c) & d) are plots for a continuous exposure.


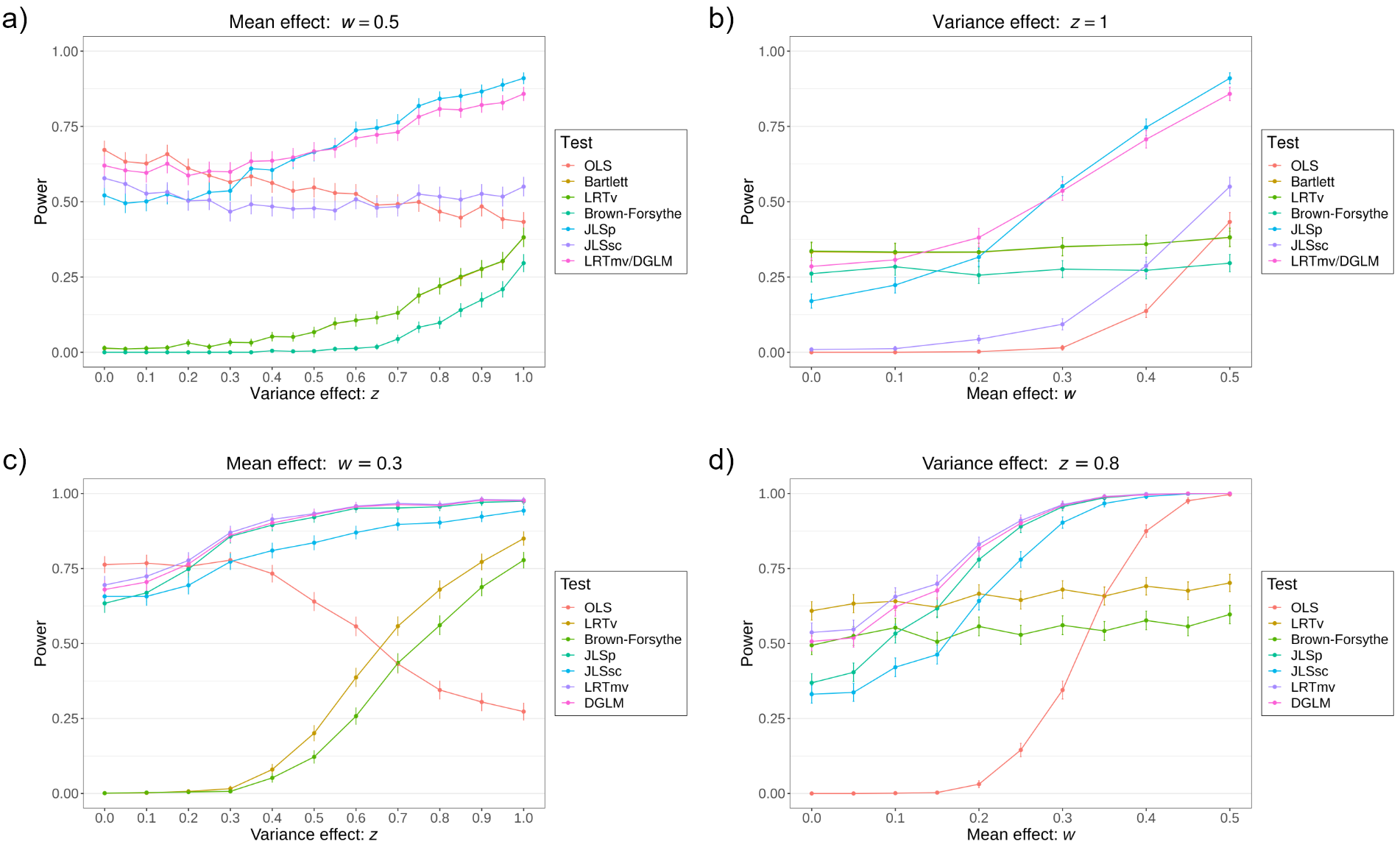


Fig. S10: Power simulation results from a skewed distribution comparing approaches for identifying CpG sites associated with either a mean and/or a variance effect with the exposure at $p<1\times{10}^{-7}$. a) & b) are plots for a binary exposure and c) & d) are plots for a continuous exposure.


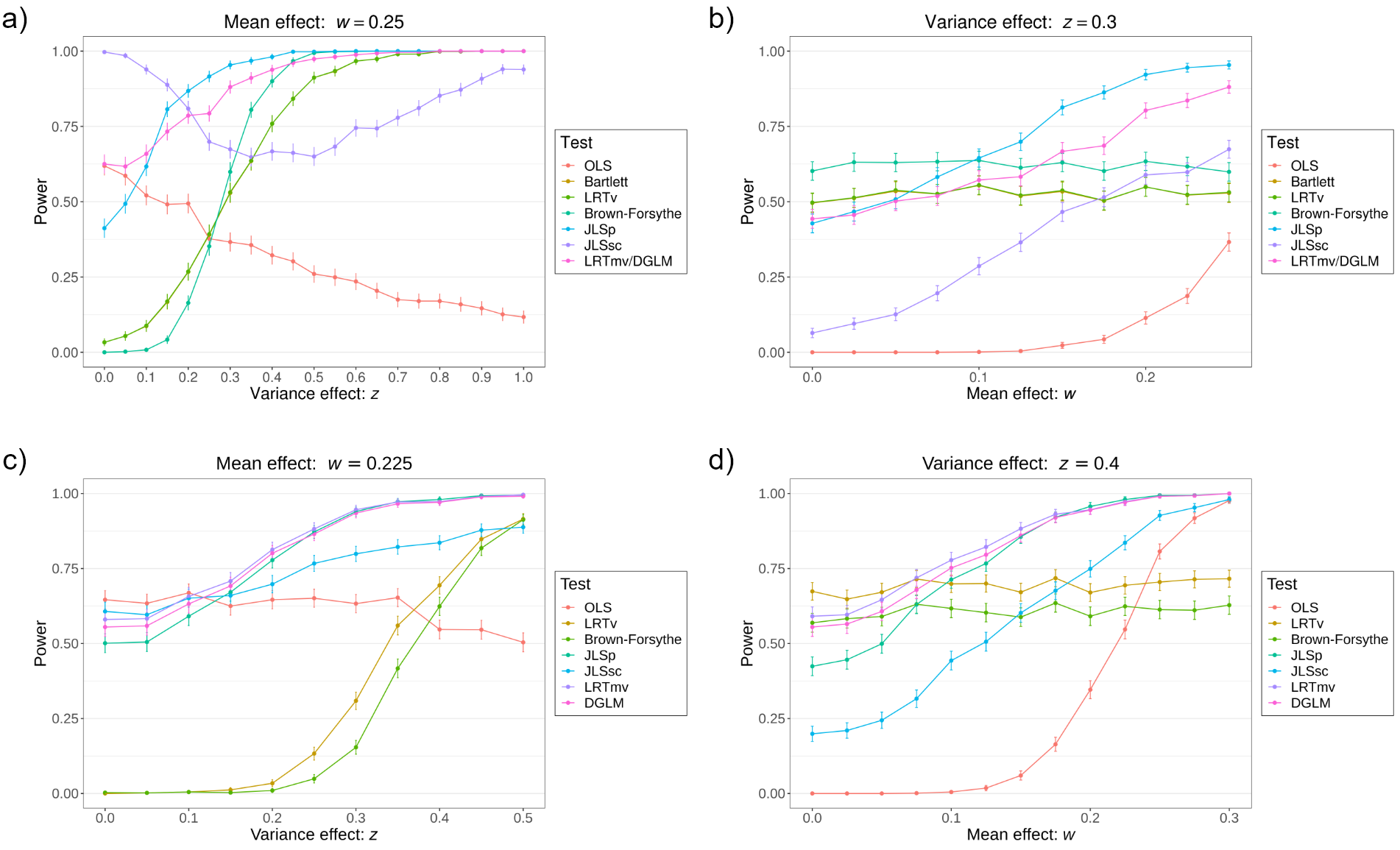


Fig. S11: The statistical properties of the JLS approaches (joint tests) for a categorical exposure with 3-levels. a) QQ plot for JLSsc for type I error simulations using 1000 samples (points that were >3×SD were defined as outliers and were removed). b) QQ plot for JLSp for type I error simulations using 1000 samples (points that were >3×SD were defined as outliers and were removed). c) Power simulations using 1000 for a mean effect of 0.4. d) Power simulations using 1000 samples for a variance effect of 0.25.


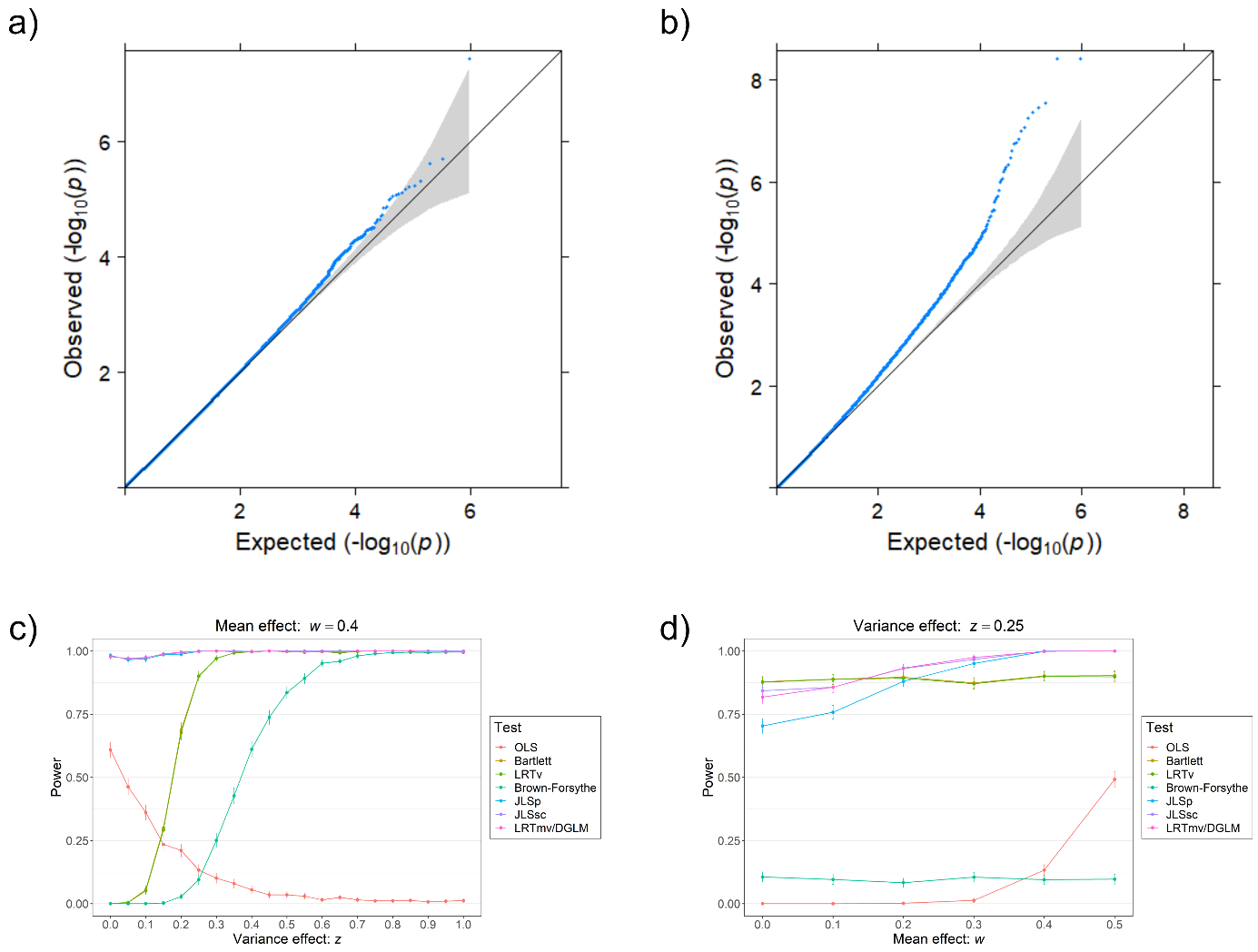


Fig. S12: The statistical properties of the JLS approaches (joint tests) in simulations including an exposure squared term for a continuous exposure. a) QQ plot for JLSsc for type I error simulations using 1000 samples. b) QQ plot for JLSsc with an $x^{2}$ term for type I error simulations using 1000 samples (points that were >3×SD were defined as outliers and were removed). c) Power simulations for a mean effect of 0.4. d) Power simulations for a variance effect of 0.7.


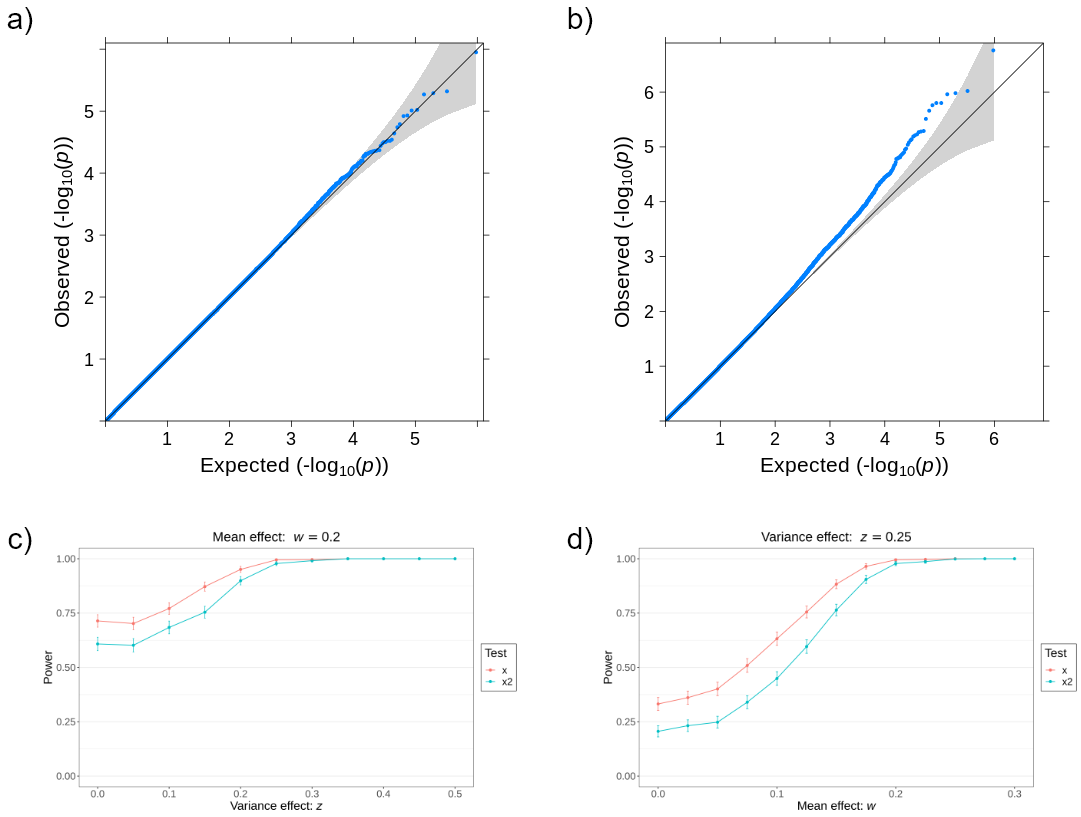


Fig. S13: The statistical properties of the JLS approaches (joint tests) in simulations with an outlier for a binary exposure. a) QQ plot for JLSsc for type I error simulations using 1000 samples. b) QQ plot for JLSp for type I error simulations using 1000 samples. c) Power simulations for a mean effect of 0.4. d) Power simulations for a variance effect of 0.7.


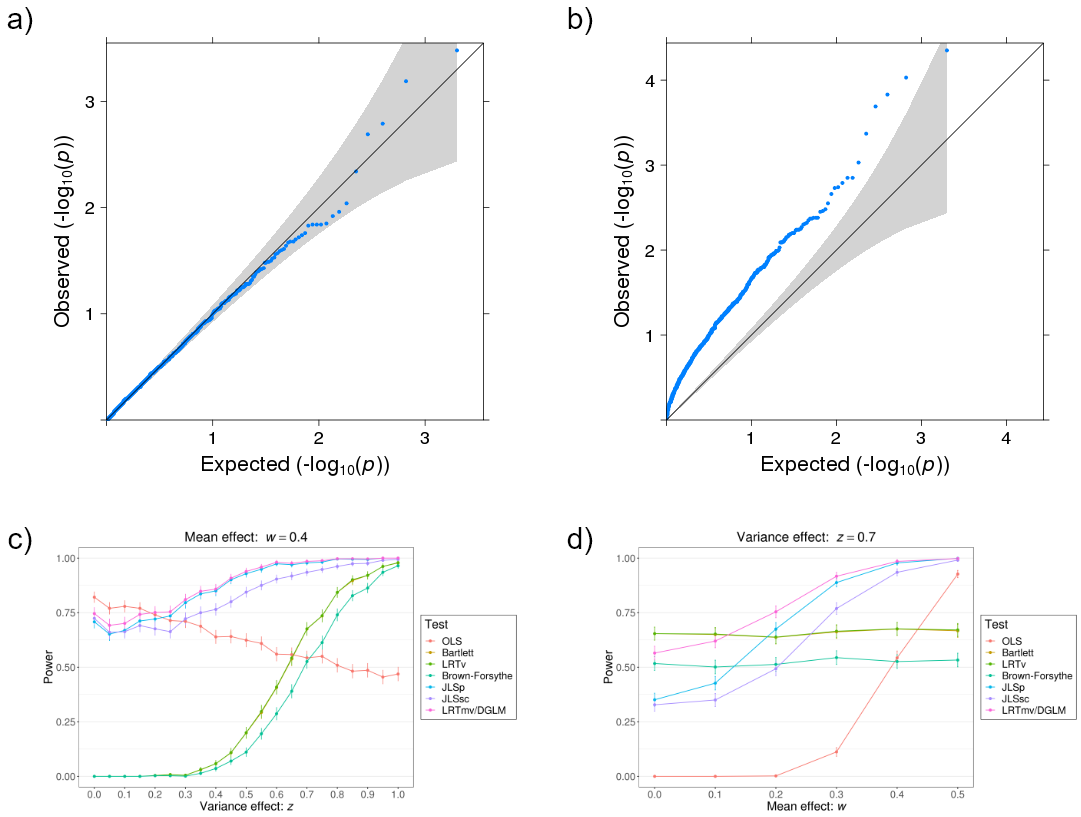


Fig. S14: The statistical properties of the JLSsc approach (joint test) using the Brown-Forsythe methodology with a binary exposure. a) QQ plot for JLSsc for type I error simulations using 1000 samples. b) QQ plot for JLSsc based on the Brown-Forsythe methodology for type I error simulations using 1000 samples c) Power simulations for JLSsc using 1000 for a mean effect of 0.4. d) Power simulations for JLSsc using 1000 samples for a variance effect of 0.7.


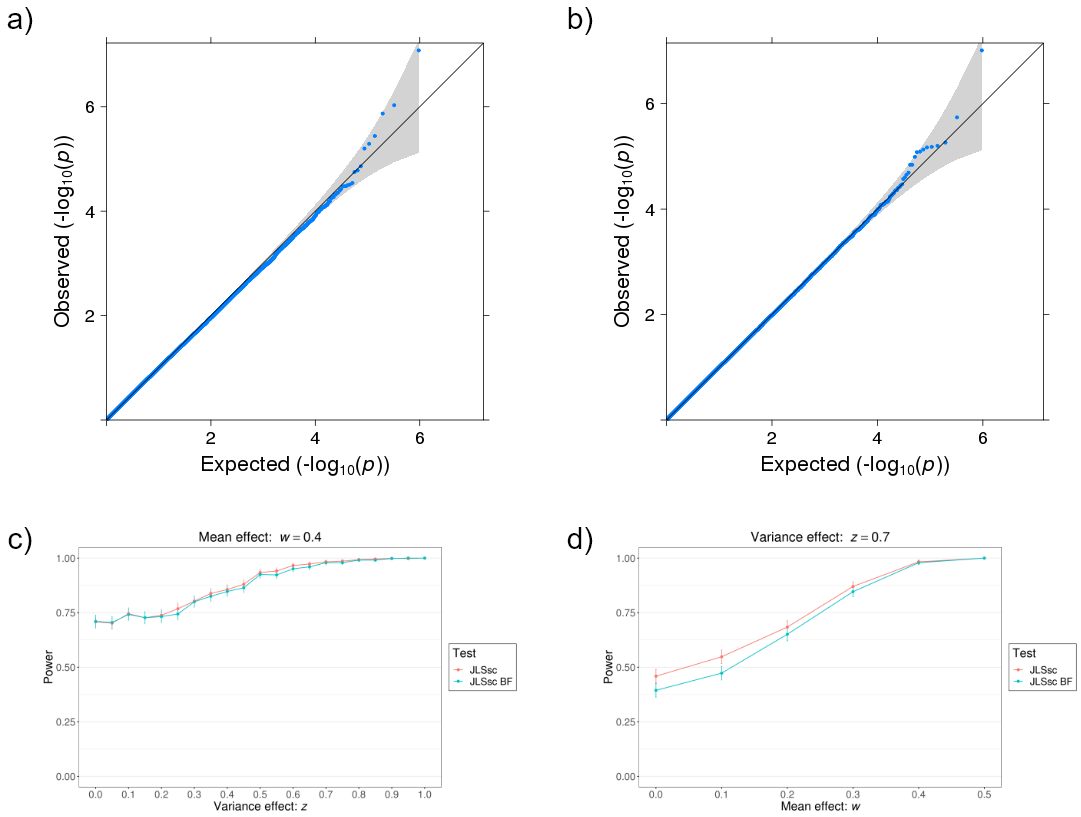


Fig. S15: QQ plots for type I error simulations using a binary exposure and 1000 repetitions. a) OLS (mean test); b) Brown-Forsythe (variability test); c) JLSp (joint test); and d) JLSsc (joint test).

Fig. S16: QQ plots for type I error simulations using a categorical (3-level) exposure and 1000 repetitions. a) OLS (mean test); b) Brown-Forsythe (variability test); c) JLSp (joint test); and d) JLSsc (joint test).

Fig. S17: QQ plots for type I error simulations using a continuous exposure and 1000 repetitions. a) OLS (mean test); b) Brown-Forsythe (variability test); c) JLSp (joint test); and d) JLSsc (joint test).

Fig. S18: The statistical properties of the JLSsc approach (joint test) relaxing the constant skewness and kurtosis assumption with a continuous exposure. a) QQ plot for JLSsc for type I error simulations using 1000 samples. b) QQ plot for JLSsc relaxing the constant skewness and kurtosis assumption for type I error simulations using 1000 samples c) Power simulations for JLSsc using 1000 for a mean effect of 0.2. d) Power simulations for JLSsc using 1000 samples for a variance effect of 0.25.


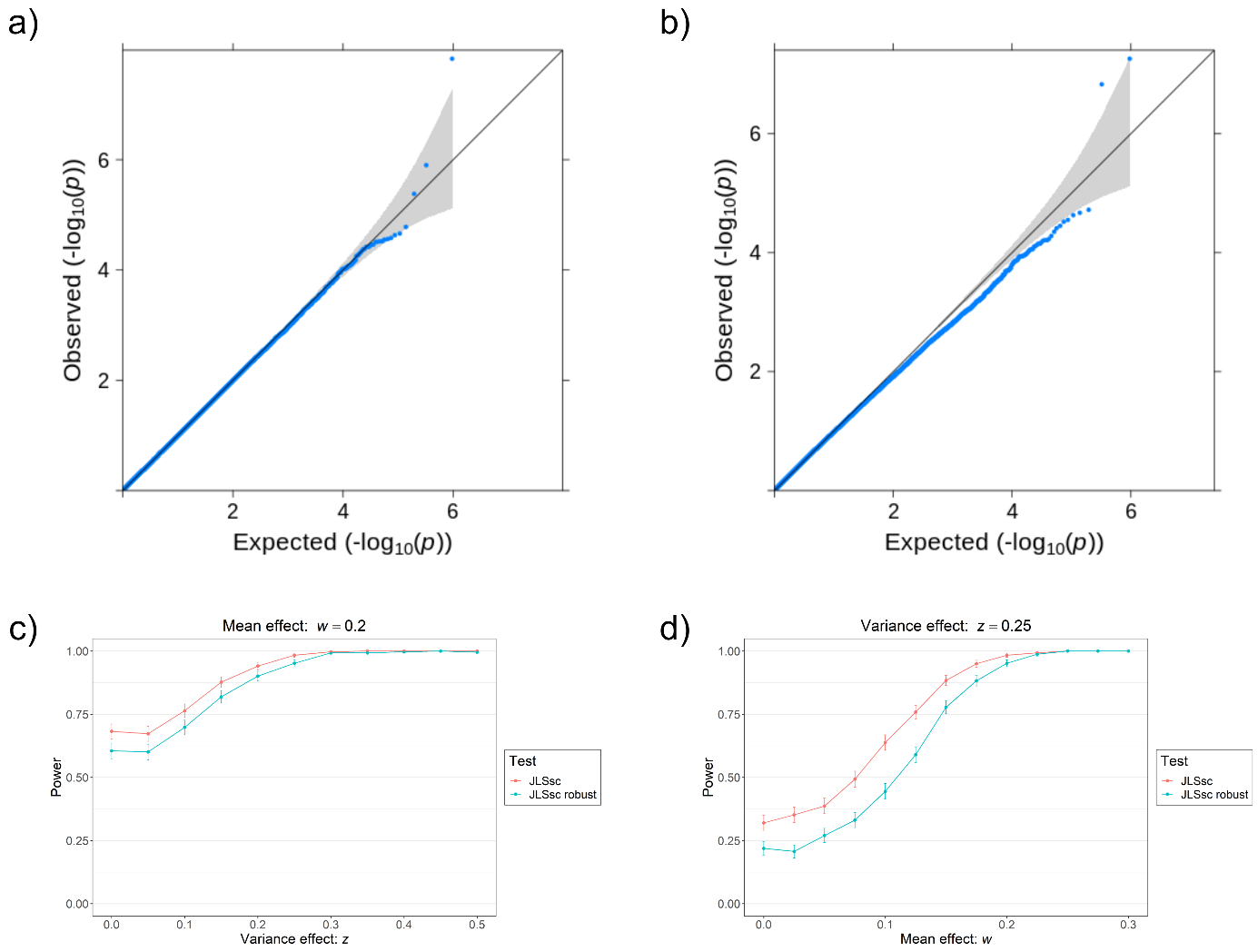


Fig. S19: The statistical properties of the JLSsc approach (joint test) relaxing the constant skewness and kurtosis assumption with a binary exposure, with no mean or variance difference but where skew and kurtosis vary by exposure. a) QQ plot for JLSsc for type I error simulations using 10,000 samples. b) QQ plot for JLSsc for type I error simulations using 10,000 samples c) QQ plot for JLSsc relaxing the constant skewness and kurtosis assumption for type I error simulations using 10,000 samples. d) QQ plot for JLSsc relaxing the constant skewness and kurtosis assumption for type I error simulations using 10,000 samples.


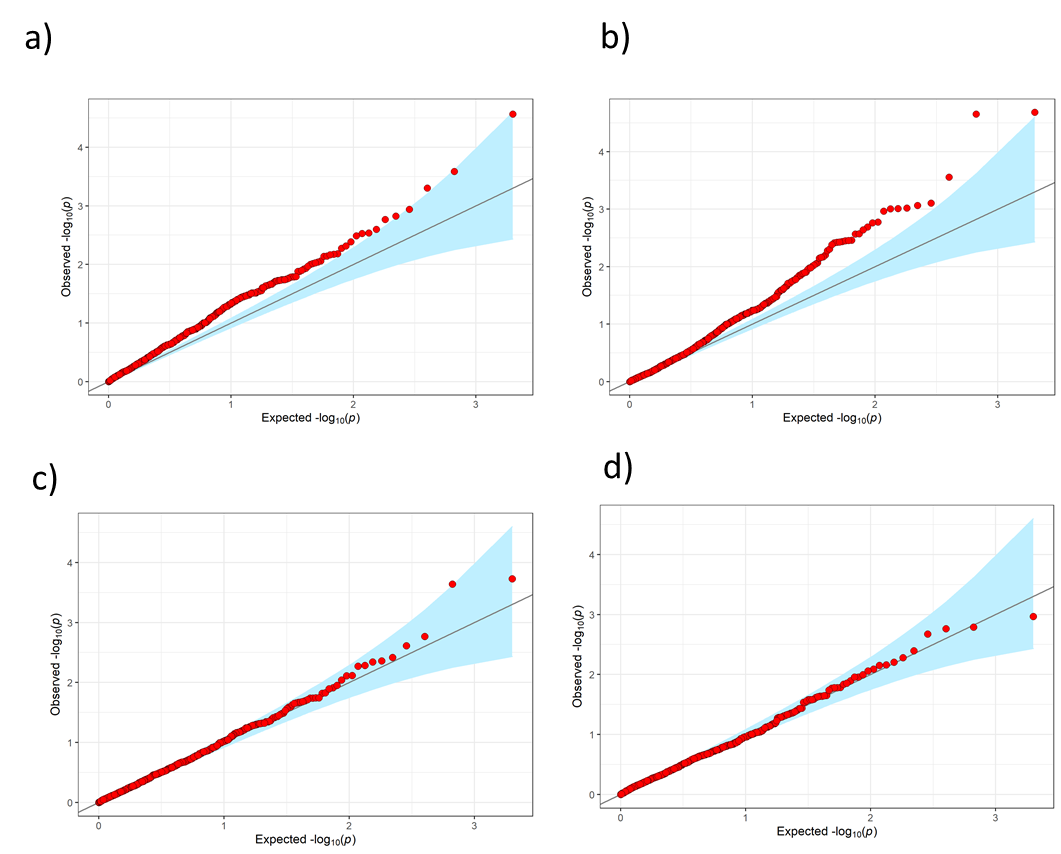


Fig. S20: The distribution of skewness a) and kurtosis b) across all 8,174 methylation sites with gender effects on methylation mean and/or variance. Dashed lines identify the 5th and 95th percentiles.


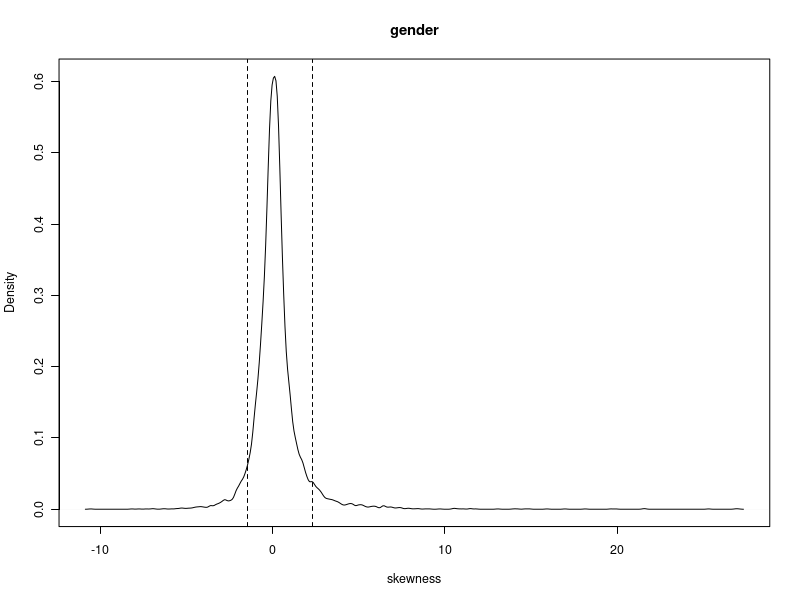


a) Skewness


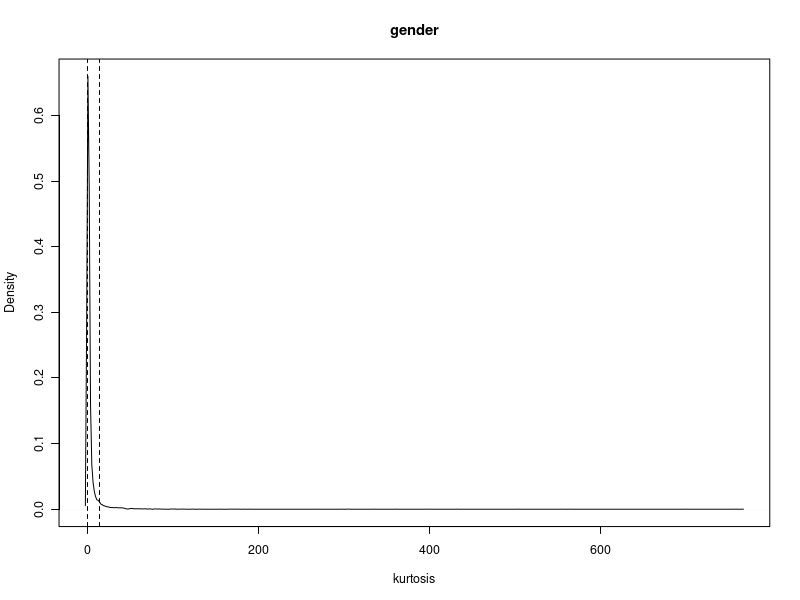


b) Kurtosis

Fig. S21: The distribution of skewness a) and kurtosis b) across all 412 methylation sites with gestational age effects on methylation mean and variance. Dashed lines identify the 5th and 95th percentiles.


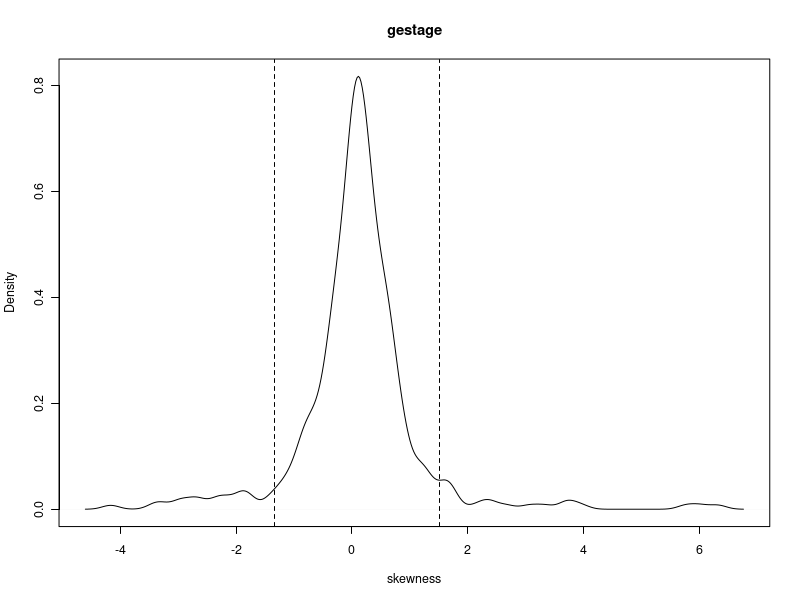


a) Skewness


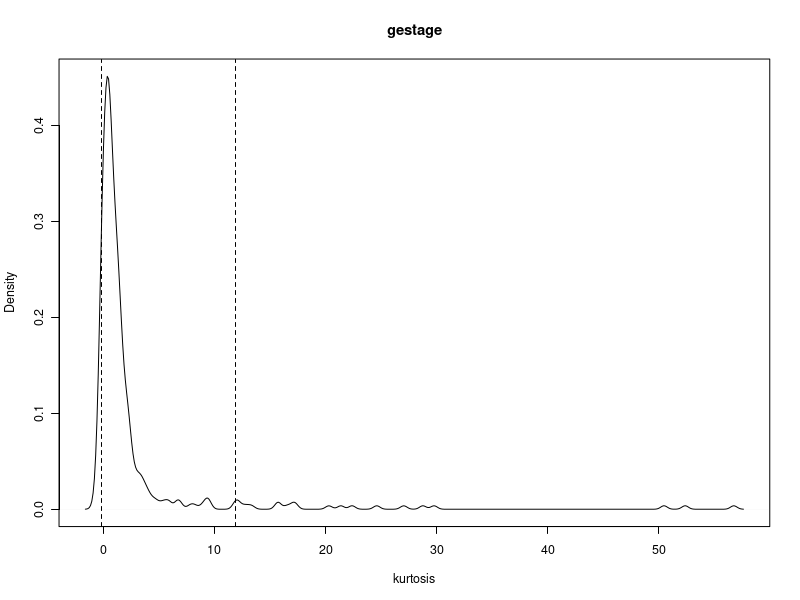


b) Kurtosis

Fig. S22: Box plot for the distribution of cg18918831 (for which there was evidence of a variance difference) by gender


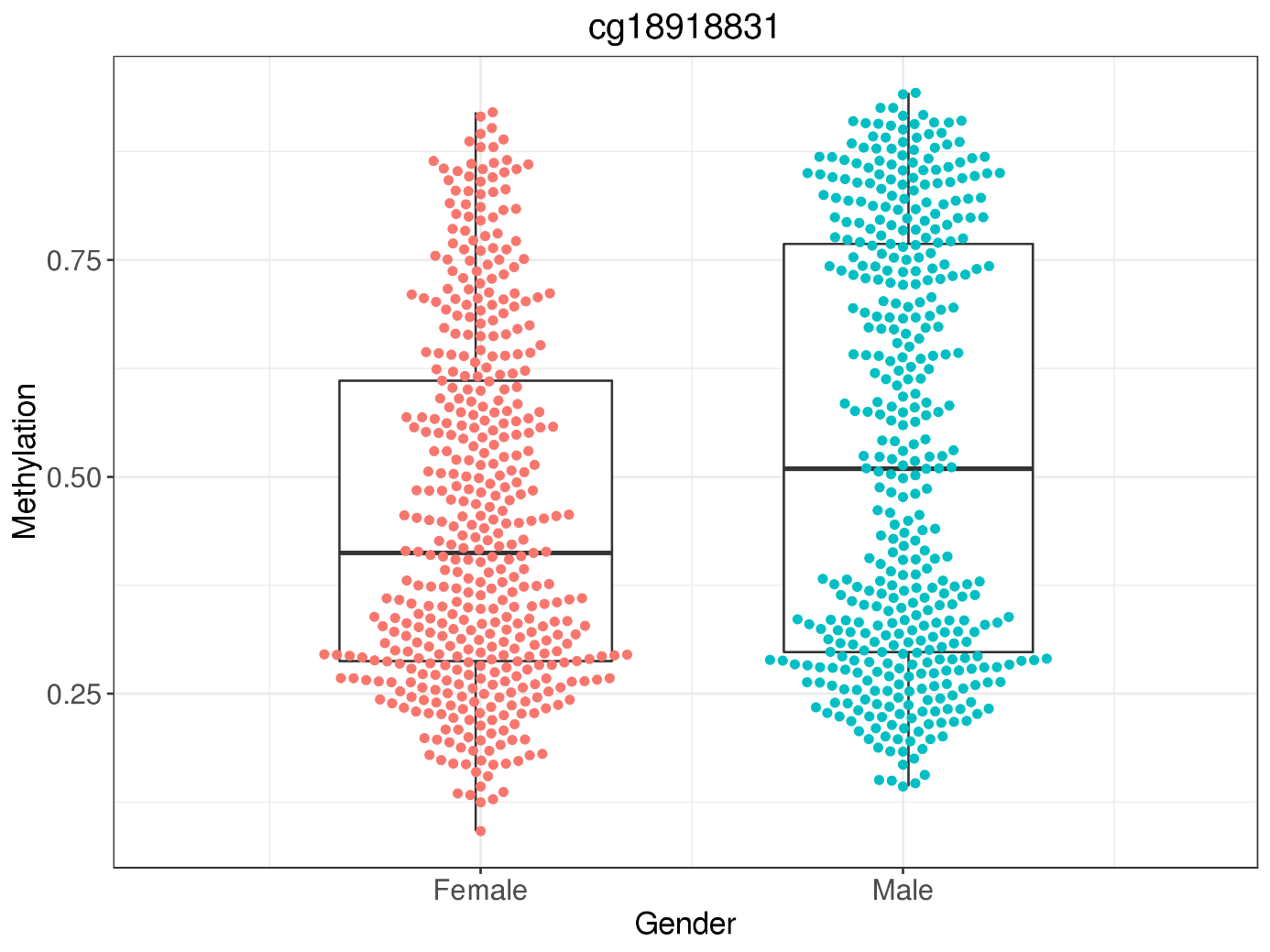

Supplement: Supplementary file 2 — Supplementary file2 (DOCX 14201 kb) [file 10654_2021_805_MOESM2_ESM.docx]
